# Depressive symptoms are associated with blunted reward learning in social contexts

**DOI:** 10.1371/journal.pcbi.1007224

**Published:** 2019-07-29

**Authors:** Lou Safra, Coralie Chevallier, Stefano Palminteri

**Affiliations:** 1 Laboratoire de Neurosciences Cognitives et Computationnelles, Institut National de la Santé et de la Recherche Médicale, Paris, France; 2 Sciences Po, CEVIPOF, CNRS, UMR7048, Paris, France; 3 Departement d’Études Cognitives, Ecole Normale Supérieure, Paris, France; 4 Université de Recherche Paris Sciences et Lettres, Paris, France; Johns Hopkins University, UNITED STATES

## Abstract

Depression is characterized by a marked decrease in social interactions and blunted sensitivity to rewards. Surprisingly, despite the importance of social deficits in depression, non-social aspects have been disproportionally investigated. As a consequence, the cognitive mechanisms underlying atypical decision-making in social contexts in depression are poorly understood. In the present study, we investigate whether deficits in reward processing interact with the social context and how this interaction is affected by self-reported depression and anxiety symptoms in the general population. Two cohorts of subjects (discovery and replication sample: *N* = 50 each) took part in an experiment involving reward learning in contexts with different levels of social information (absent, partial and complete). Behavioral analyses revealed a specific detrimental effect of depressive symptoms–but not anxiety–on behavioral performance in the presence of social information, i.e. when participants were informed about the choices of another player. Model-based analyses further characterized the computational nature of this deficit as a negative audience effect, rather than a deficit in the way others’ choices and rewards are integrated in decision making. To conclude, our results shed light on the cognitive and computational mechanisms underlying the interaction between social cognition, reward learning and decision-making in depressive disorders.

## Author summary

Blunted sensitivity to rewards is at the core of depression. However, studies that investigated the influence of depression on decision-making have often done so in asocial contexts, thereby providing only partial insights into the way depressive disorders impact the underlying cognitive processes. Indeed, atypical social functioning is also a central characteristic of depression. Here, we aimed at integrating the social component of depressive disorders into the study of decision-making in depression. To do so, we measured the influence of self-reported depressive symptoms on social learning in participants performing an online experiment. Our study shows that depressive symptoms are associated with decreased performance only when participants are informed about the actions of another player. Computational characterizations of this effect reveal that participants with more severe depressive symptoms differ only in the way they learn from their own actions in a social context. In other words, our results indicate that depressive symptoms are associated with a negative audience effect and thus provide new insights into the way social cognition and decision-making processes interact in depression.

## Introduction

One of the core clinical symptoms of depression is anhedonia, which refers to a reduced motivation to engage in daily life activities (motivational anhedonia) and a reduced enjoyment of usually enjoyable activities (consummatory anhedonia) [*1*, *2*]. In principle, this clinical manifestation could be explained by reduced reward sensitivity, both in terms of incentive motivation and in terms of reinforcement processes [*3*–*5*]. A direct prediction of this hypothesis is that depressive symptoms should be associated with reduced reward sensitivity in learning contexts both at the behavioral and neural level. However, while some studies do find evidence that depressive symptoms in the general population and in clinical depression are associated with blunted reward learning and reward-related signals in the brain [*6*, *7*], others indicate no [*8*, *9*] or mixed effects [*5*]. As a consequence, there is no strong consensus about which components of reward processing are most predictive of depressive symptoms in both the general population and clinical depression [*5*].

Another striking clinical manifestation of depressive symptoms is a marked decrease in social interactions. Depression is indeed associated with social risk factors, social impairments and poor social functioning [*10*]. Surprisingly, despite the importance of the socio-cognitive impairments that are often associated with elevated depressive symptoms, non-social aspects have received disproportionate attention. Furthermore, when social aspects are investigated the focus is often on emotional processing and theory of mind but not on how social information is integrated to produce efficient goal-directed behavior [*11*]. In the present study, our goal was to investigate whether the reward-learning deficit that is often associated with elevated depressive symptoms interacts with the social context [*12*].

**Fig 1:**
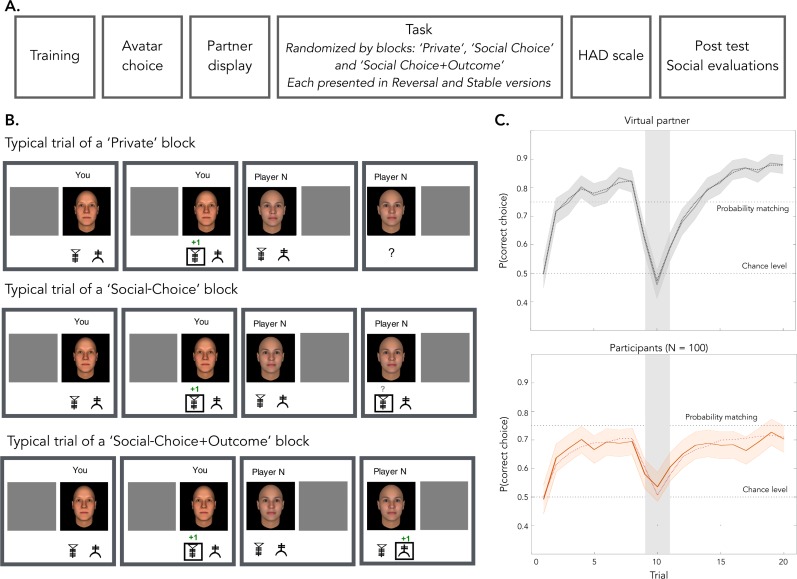
**Learning task and learning behavior (A) Experimental procedure.** Participants first performed a training session before choosing their avatar for the task. They were then paired with another player (simulated) represented by an avatar neutral in trustworthiness and dominance. Participants then performed the behavioral task that were organized by randomized blocks. Each block corresponded to a learning condition ‘Private’, ‘Social-Choice’ or ‘Social-Choice+Outcome’ presented once with stable contingencies and one with unstable contingencies (reversal condition). After the task, participants completed the HAD questionnaire and performed the social evaluations as a post-test. **(B) Behavioral task.** In each condition, participants played in turn with a virtual demonstrator. In each private trial, after each choice, participants received a reward or a punishment. In the Private blocks, participants did not see the choice or the outcome of the demonstrator. In the Social-Choice blocks, the choice of their demonstrator was displayed at each trial. In the Social-Choice+Outcome blocks, both the choice and the outcome of the demonstrator were displayed. **(C) Learning behavior of the virtual demonstrator and the participants.** The behavior of the virtual partner (top) was simulated using a reinforcement learning model (whose parameters were correctly recovered by our model optimization procedure: black dotted line). Participants accurately learned which option was the most rewarded across the trial. In both the real and simulated tasks a reversal of the contingencies occured at the 10^th^ ±1 trial (grey shaded area).

According to social learning theory, a sizable amount of decisions are not directly shaped by people’s personal history of reward and punishments, but are rather acquired through social observation [*13*]. More specifically, this framework posits that human learning occurs mostly in social contexts, where subjects can be influenced by social cues (i.e. others’ choices and outcomes) [*13*, *14*]. In order to test how depressive symptoms affect the integration of social cues during reinforcement learning, we administered a variant of a previously validated observational learning task on two independent samples of participants [*14*, *15*]. Subjects also completed psychometric questionnaires assessing depression and anxiety (a co-morbid trait) symptoms. The task included a ‘Private’ learning condition, in which participants only had access to the outcome of their own choice, and two social conditions: the ‘Social-Choice’ condition in which participants had access to the demonstrator’s choice, and the ‘Social-Choice+Outcome’ condition in which participants had access to the demonstrator’s actions and their outcome (**Fig 1A and 1B**).

Our design allowed us to test several hypotheses concerning the relation between depressive symptoms and learning performance in private and social contexts. First, our design allowed us to test whether or not depressive symptoms degrade reward learning *per se*, as assumed by the standard account of depression as a reward sensitivity deficit. Second, by comparing the ‘Private’ and the ‘Social’ learning contexts, we could assess whether or not depressive symptoms are associated with a learning deficit in ‘Social’ contexts, as predicted by evidence of socio-cognitive impairments in depressive patients. Finally, thanks to computational analyses, we could precisely characterize the learning deficit in the ‘Social’ context either as a primary social learning deficit (i.e. impaired imitation) or as a secondary social learning (i.e. a negative audience effect).

## Results

### Experimental protocol and quality checks

An online experiment was particularly suited to test our hypothesis because—compared to laboratory-based experiments—it provides a more diversified pool of subjects, in terms of psychiatric traits and cognitive performance [*16*–*19*]. Specifically, we tested 50 participants in the general population and then ran a direct replication of the experiment on a second independent sample of 50 participants. In the main text, we report the meta-analytical p-values computed using a mixed effect meta-analysis. In the tables we present the results separately for each experiment and highlight the replication criteria proposed by the open science framework [*20*].

Levels of depressive and anxiety symptoms spanned a large range (**Table 1**) [*21*], with good internal consistency (Hospital Anxiety Depression scale—depression subscale: Cronbach’s alpha 85%; anxiety subscale: Cronbach’s alpha 84%). Participants were paired with a virtual demonstrator and performed a probabilistic reinforcement learning task in three contexts: a ‘Private’ condition, in which participants performed the task individually with no access to the demonstrator’s choices and outcomes, and two social conditions: the ‘Social-Choice’ condition in which participants had access to the demonstrator’s choices, and the ‘Social-Choice+Outcome’ condition in which participants had access to the demonstrator’s choices and their outcome. Overall, participants displayed robust instrumental learning and chose the most rewarded symbol above chance in all conditions (meta-analysis ‘Private’: *M*_*META*_ = 0.65 ± 0.03, *z*_*META*_ = 11.37, *p* < .001; ‘Social-Choice’: *M*_*META*_ = 0.65 ± 0.03, *z*_*META*_ = 11.83, *p* < .001; ‘Social-Choice+Outcome’: *M*_*META*_ = 0.67 ± 0.03, *z*_*META*_ = 12.45, *p* < .001; ± corresponds to the 95% confidence intervals; **Fig 1C**; See **S1 Table** for the results on the two samples separately).

**Table 1:** Descriptive statistics for age, gender, depression and anxiety scores. For each sample, the mean of each demographic variable is presented with its 95% confidence interval.

	Age	Sex ratio (% women)	Depression scores	Anxiety scores	Correlation between Depression and Anxiety scores
Discovery sample (N = 50)	33.02 ± 1.25 [22–62]	28%	5.46 ± 1.26 [0–19]	6.40 ± 1.16 [0–15]	r = .44, t(48) = 3.43, p = .001
Replication sample (N = 50)	33.76 ± 3.28 [19–61]	42%	4.96 ± 1.27 [0–16]	6.30 ± 1.28 [0–20]	r = .74, t(48) = 7.61, p < .001
Statistical difference	t(98) = 0.36 p > .250	X-squared = 1.58, df = 1, p-value = .208	t(98) = 0.56 p > .250	t(98) = 0.12 p > .250	

### Assessing observational learning

**Fig 2:**
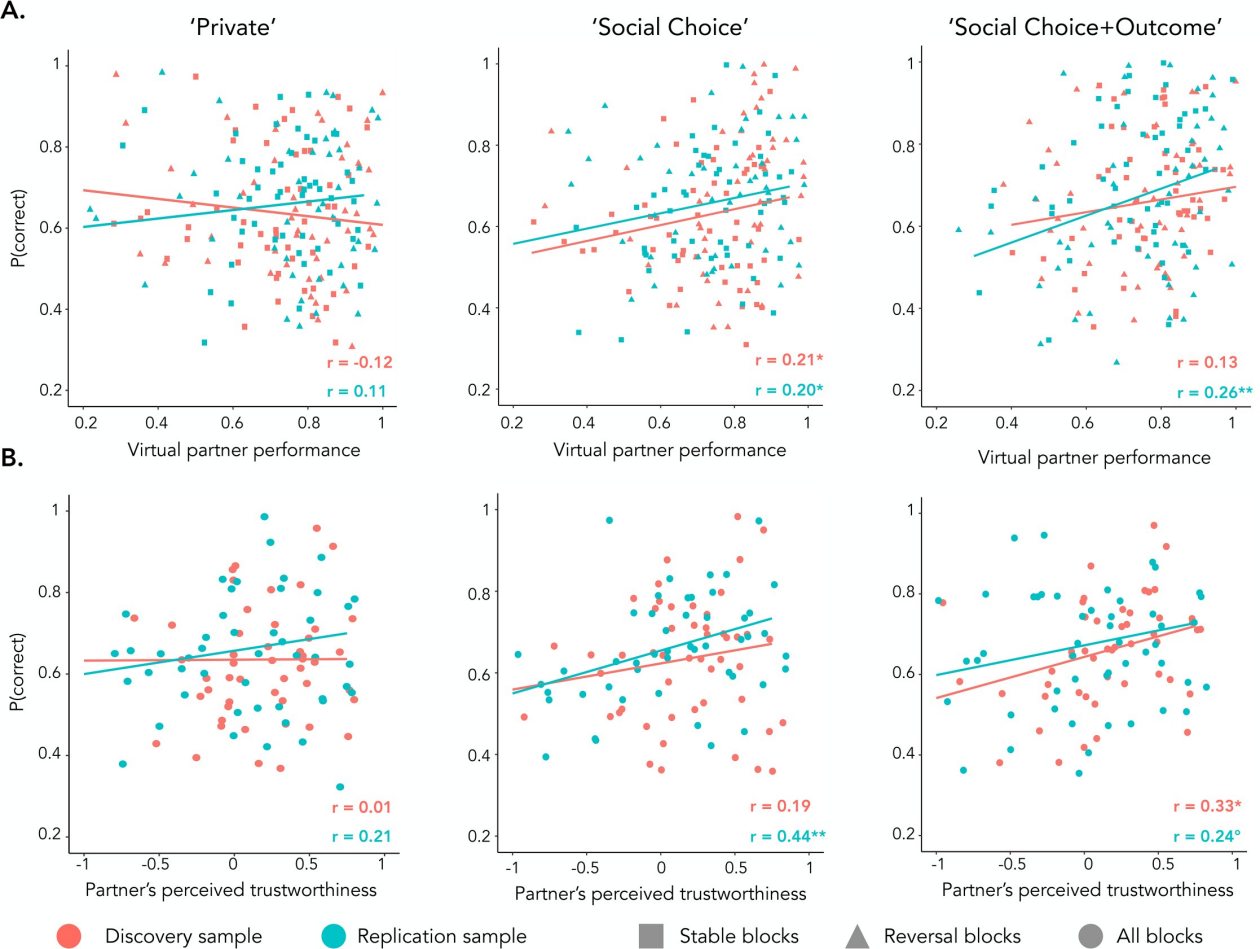
**Assessing social reinforcement learning (A) Effect of demonstrator’s behavior.** Scatter plots representing the correlation between the correct choice rate and the performance of the demonstrator in the three learning contexts (from left to right: ‘Private’, ‘Social Choice’, ‘Social Choice+Outcome’). **(B) Effect of perceived trustworthiness**. Scatter plots representing the correlation between the correct choice rate and the reported trustworthiness in the three learning contexts. ‘r’ = Pearson’s correlation coefficient. °p<0.10, *p<0.05, **p<0.01, Pearson’s correlation.

Contrary to previous studies [*14*, *15*], we used an online adaptive learning algorithm that determined the demonstrator’s behavior (Q-learning with learning rate = 0.5 and choice temperature = 10). As a consequence, the virtual demonstrators displayed realistic learning curves with some variability of performance (**Fig 1C**). We predicted that observational learning would result in a correlation between the participants’ and the demonstrator’s correct choice rate in a given learning session. As predicted, a higher correct choice rate for the demonstrator was associated with a higher correct choice rate for participants in both social conditions (‘Social-Choice’ condition: *r*_*META*_ = .20 ± 0.07, *z*_*META*_ = 2.89, *p* = .004; ‘Social-Choice+Outcome’ condition: *r*_*META*_ = .20 ± 0.07, *z*_*META*_ = 2.87, *p* = .004) but not in the private condition (*r*_*META*_ = -.01 ± 0.11, *z*_*META*_ = -0.05, *p* > .250; **Fig 2A**; see **Table 2** for the results on the two samples separately).

**Table 2:** Main statistical effects obtain by correlations on the performances in ‘Private’, ‘Social-Choice’ and ‘Social-Choice+Outcome’ conditions, with three replication criteria. For each correlation we report the result (Pearson’s correlation coefficient, p-value and t-value; (± corresponds to s.e.m.) in the first (E0) and the second (E1) experiment, as well as the meta-analytical p-value (E_META_). For the results with a significant meta-analytical p-value, to better visualize the replicability, we also explicitly report replication parameters (‘+’ = yes; ‘-‘ = no): i) whether or not the E1 effect is within the 95% confidence interval of the E0 effect; ii)whether or not the effect was significant in both experiments; (iii) whether or not E_META_ was significant. n.a.: not applicable.

	Discovery sample (E_0_)	Replication sample (E_1_)	Meta-analysis (E_meta_)	E_1_ ∈ E_0_ ± 95% conf. interval	E_0_ significant & E_1_ significant	E_meta_ significant
**Effect of virtual demonstrator’s performance on performance**						
‘Private’ condition	r = -.12 ± .10 t(98) = -1.16 p = .247	r = .11 ± .10 t(98) = 1.06 p = .292	r = -.01±.11 z = -0.05 p > .250	na	na	na
‘Social Choice’ condition	r = .21 ± .10 t(98) = 2.10 p = .039	r = .20± .10 t(98) = 1.20 p = .049	r = .20 ± .07 z = 2.89 p = .004	**+**	**+**	**+**
‘Social Choice+Outcome’ condition	r = .13 ± .10 t(98) = 1.34 p = .182	r = .29+.10 t(98) = 2.69 p = .008	r = .20 ±.07 z = 2.87 p = .004	**+**	**-**	**+**
**Effect of virtual demonstrator’s perceived trustworthiness on performance**						
‘Private’ condition	r = .01 ± .14 t(48) = .95 p > .250	r = .21± .14 t(48) = 1.50 p = .141	r = .11± .10 z = 1.09 p > .250	na	na	na
‘Social Choice’ condition	r = .18 ± .14 t(48) = 1.33 p = .189	r = .44 ± .13 t(48) = 3.42 p = .001	r = .32 ± .13 z = 2.54 p = .011	**+**	**-**	**+**
‘Social Choice+Outcome’ condition	r = .33 ± .14 t(48) = 2.42 p = .019	r = .24 ± .14 t(48) = 1.65 p = .087	r = .29 ± .10 z = 2.96 p = .003	**+**	**-**	**+**
**Effect of depression scores on performance**						
‘Private’ condition	r = .19 ± .14 t(48) = 1.31 p = .198	r = -.14 ± .14 t(48) = -0.95 p > .250	r = .04± .16 z = 0.16 p > .250	na	na	na
‘Social Choice’ condition	r = -.30 ± .14 t(48) = -2.15 p = .036	r = -.36 ± .13 t(48) = -2.75 p = .008	r = -.33± .10 z = -3.47 p < .001	**+**	**+**	**+**
‘Social Choice+Outcome’ condition	r = -.08 ± .14 t(48) = -0.58 p > .250	r = -.01± .14 t(48) = -0.10 p > .250	r = -.05 ± .10 z = -0.48 p > .250	na	na	na
**Effect of anxiety scores on performance**						
‘Private’ condition	r = -.02 ± .14 t(48) = -0.02 p > .250	r = -.05 ± .14 t(48) = -0.33 p > .250	r = -.03 ± .10 z = -0.34 p > .250	na	na	na
‘Social Choice’ condition	r = -.24 ± .14 t(48) = -1.68 p = .099	r = -.13 ± .14 t(48) = -0.92 p > .250	r = -0.18 ± .10 z = -1.85 p = .065	na	na	na
‘Social Choice+Outcome’ condition	r = -.29 ± .14 t(48) = -2.13 p = .038	r = 0.01 ± .14 t(48) = 0.12 p > .250	r = -0.14 ± .16 z = -0.91 p > .250	na	na	na
**Effect of depression scores on learning parameters**						
Temperature ‘Private’ condition (ß_P_)	r = .06 ± .14 t(48) = 0.42 p > .250	r = -.15 ± .14 t(48) = -1.06 p > .250	r = -.05 ± .11 z = -0.44 p > .250	na	na	na
Learning rate ‘Private’ condition (α_P_)	r = -.09 ± .15 t(48) = -0.64 p > .250	r = .26 ± .14 t(48) = 1.14 p > .250	r = .04 ± .13 z = 0.29 p > .250	na	na	na
Temperature ‘Social’ conditions (ß_S_)	r = .02 ± .14 t(48) = 0.13 p > .250	r = -.13 ± .14 t(48) = -0.88 p > .250	r = -.05 ± .10 z = -0.53 p > .250	na	na	na
Learning rate ‘Social’ conditions (α_S_)	r = -.17 ± .14 t(48) = -1.32 p = .194	r = -.31 ± .13 t(48) = -2.28 p = .028	r = -.25 ± .10 z = -2.54 p = .011	**+**	**-**	**+**
Action imitation parameter (κ)	r = .00 ± .14 t(48) = 0.02 p > .250	r = -.15 ± .14 t(48) = -1.06 p > .250	r = -.08 ± .10 z = -0.74 p > .250	na	na	na
Social learning parameter (α_O_)	r = -.20 ± .14 t(48) = -1.43 p = .158	r = -.06 ± .14 t(48) = -0.40 p > .250	r = -.13 ± .10 z = -1.30 p = .193	na	na	na

In order to confirm that participants actually integrated the virtual demonstrator as a social partner, we measured the influence of participants’ rating of trustworthiness of the demonstrator’s face on social learning. An effect of perceived trustworthiness evaluations was found, such that participants who perceived the demonstrator’s avatar as more trustworthy had higher correct choice rates in the ‘Social-Choice’ (*r*_*META*_ = .32 ± 0.13, *z*_*META*_ = 2.54, *p* = .011) and in the ‘Social-Choice+Outcome’ conditions (*r*_*META*_ = .29 ± 0.10, *z*_*META*_ = 2.96, *p* = .003) but not in the ‘Private’ condition (*r*_*META*_ = .11 ± 0.10, *z*_*META*_ = 1.09, *p* > .250; **Fig 2B**). This effect of the social evaluation of the demonstrator’s avatar confirms that participants processed the information in a social context.

### Correlation between depressive symptoms and performance

**Fig 3:**
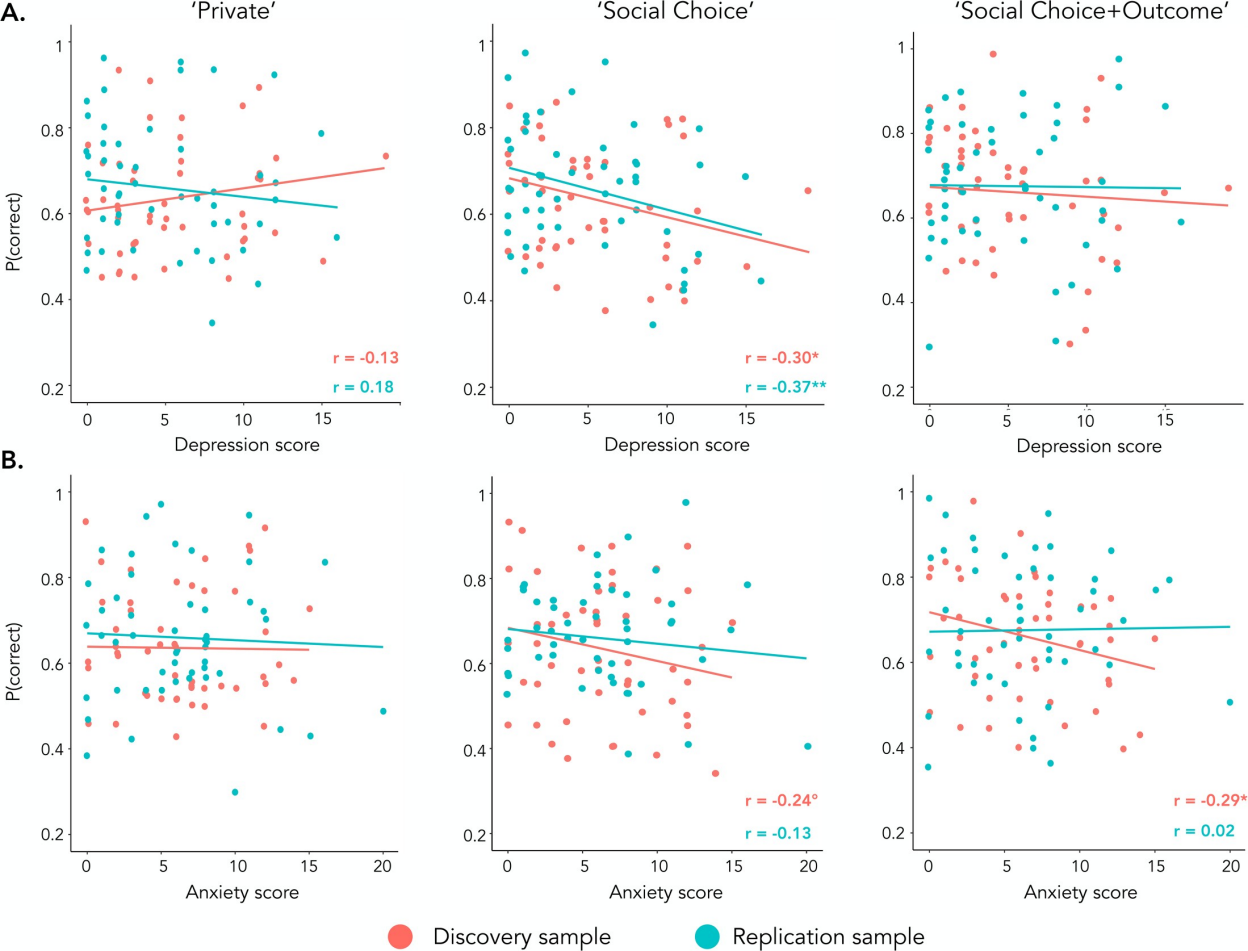
Effect of depression scores on reinforcement learning. **(A) Effect of depression scores on learning.** Scatter plots representing the correlation between the correct choice rate and the self-reported depression score in the three learning contexts (from left to right: ‘Private’, ‘Social Choice’, ‘Social Choice+Outcome’). **(B) Effect of anxiety scores on learning.** Scatter plots representing the correlation between the correct choice rate and the self-reported anxiety score in the three learning contexts ‘r’ = Pearson’s correlation coefficient. °p<0.10, *p<0.05, Pearson’s correlation.

A significant effect of depressive symptoms was found such that the higher the depressive symptoms, the lower the rate of correct choices in the ‘Social-Choice’ condition only (*r*_*META*_ = -.33 ± 0.10, *z*_*META*_ = -3.47, p < .001; ‘Private’ condition: *r*_*META*_ = .04 ± 0.16, *z*_*META*_ = 0.16, p > .250; ‘Social-Choice+Outcome’ condition: *r*_*META*_ = -.05 ± 0.10, *z*_*META*_ = -0.48, p > .250; **Fig 3A**). However, a similar effect of anxiety, which is a comorbid trait of depression [*22*, *23*], was found as a trend (*r*_*META*_ = -0.18 ± 0.10, *z*_*META*_ = -1.85, *p* = .065; **Fig 3B**). In order to better understand the effect of depressive symptoms on learning in social contexts, we ran a mixed linear logistic regression that included depressive and anxiety scores, taken as continuous between-subject variables (the regression also included a range of controls listed in **Table 3**). The analysis revealed a significant effect of depression scores such that the higher the depressive scores, the lower the rate of correct choices in the ‘Social-Choice’ condition compared to the ‘Private’ condition (*z*_*META*_ = -2.85, *p* = .004; no other significant effect of depression and anxiety scores was evidenced: all *p*s > .250; **Fig 3A**). Importantly, the negative effect of depressive symptoms in the ‘Social-Choice’ condition was particularly robust, because it was found in both the discovery and the replication sample and in the blocks with stable and reversal contingencies (within-subject) (**S2 Fig**).

**Table 3:** Effects (mixed linear model) of social information (‘Social-Choice’ and ‘Social-Choice+Outcome’), virtual demonstrator correct choice rate, perceived trustworthiness (‘Trustworthiness’), HAD scores (‘Depression’ and ‘Anxiety’), and their interactions compared to the ‘Private’ condition. °p<0.10, *p<0.05, **p<0.01, z-test.

Effect	Coefficient	SEM	z-value	P-value
Intercept	0.15	0.05	2.90	.005**
Social-Choice	-0.13	0.07	-1.95	.052°
Social-Choice+Outcome	-0.13	0.07	-1.70	.089*
Demonstrator performance	-0.01	0.07	-0.09	.925
Trustworthiness	0.03	0.03	0.89	.372
Depressive symptoms	0.00	0.01	0.50	.615
Anxiety symptoms	-0.00	0.00	-0.40	.690
Social-Choice x Demonstrator performance	0.23	0.09	2.72	.007**
Social-Choice+Outcome x Demonstrator performance	0.21	0.11	2.00	.045*
Social-Choice x Trustworthiness	0.05	0.03	1.53	.127
Social-Choice+Outcome x Trustworthiness	0.06	0.05	1.35	.176
Social-Choice x Depressive symptoms	-0.01	0.00	-2.85	.004**
Social-Choice+Outcome x Depressive symptoms	-0.00	0.00	-0.83	.407
Social-Choice x Anxiety symptoms	0.00	0.00	0.52	.604
Social-Choice+Outcome x Anxiety symptoms	-0.00	0.00	-0.85	.398

**Fig 4:**
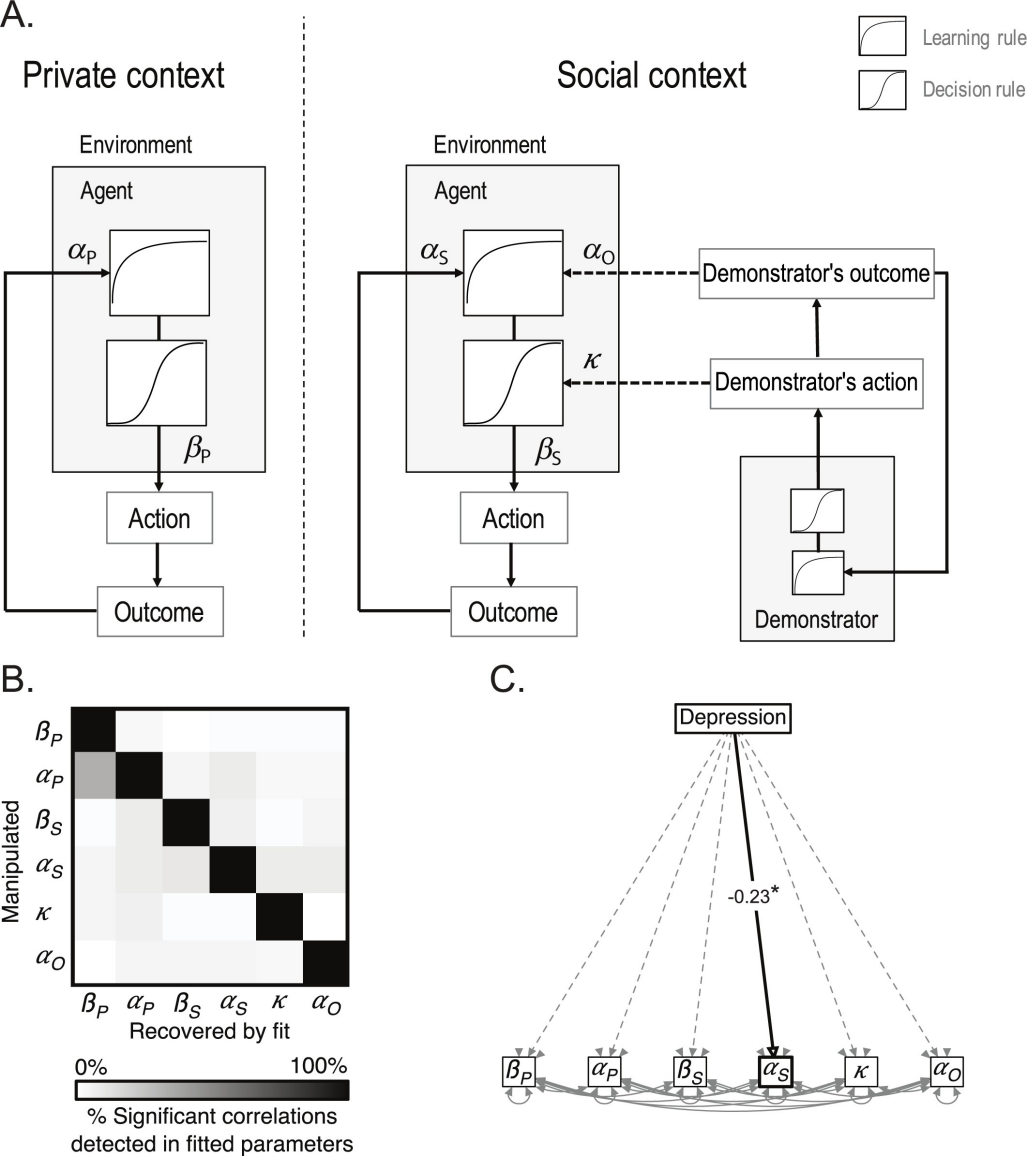
**Social reinforcement learning model (A) Computational model.** A social reinforcement learning model was fitted on participants’ behavior. In the ‘Private’ condition (‘Private context’), the model corresponded to a classical Q-learning (or Rescorla-Wagner) model. In Social context’ (‘Social-Choice’ and ‘Social-Choice+Outcome’ conditions), the model assumes that social information is integrated into the learning and decision process. Following Burke et al. [*14*], choice probability was updated based on the demonstrator’s action (imitation) in the ‘Social-Choice’ condition and the option value was updated when the demonstrator’s outcome was presented (counterfactual learning) in the ‘Social-Choice+Outcome’ condition. The proposed model also allows for different private parameters (learning rate, *α*_*S*_, and choice randomness, *β*_*S*_) being in the Social context. **(B) Parameter recovery.** To assess the sensitivity and the specificity of our model fitting procedure, we conducted a parameter recovery analysis. The matrix represents the percentage of significant correlations detected between different combinations of parameters. The diagonal cases correspond to the correlations that are accurately recovered; the other cases correspond to correlations that are spuriously recovered. **(C) Effect of depression on the model parameters.** Depression was specifically associated with a decrease in the private learning rate in the Social context α_S_), even controlling for the correlation between the different model parameters (structural equation modeling).

Finally, we tested whether the correct choice rates in the ‘Social-Choice’ condition identified participants with difficulties linked to depressive symptoms (i.e. scoring ≥ 8 on the HAD depression subscale [*21*]) from participants in whom these difficulties are absent. The classification analysis revealed that the performance in the ‘Social-Choice’ condition identified participants with depressive symptoms with good accuracy of 73 ± 1% and with good sensitivity, or True Positive Rate (82 ± 2%) but low specificity, or True Negative Rate (53 ± 3%) of the classifier (**Fig 4A**).

### Computational model-based analyses

**Fig 5:**
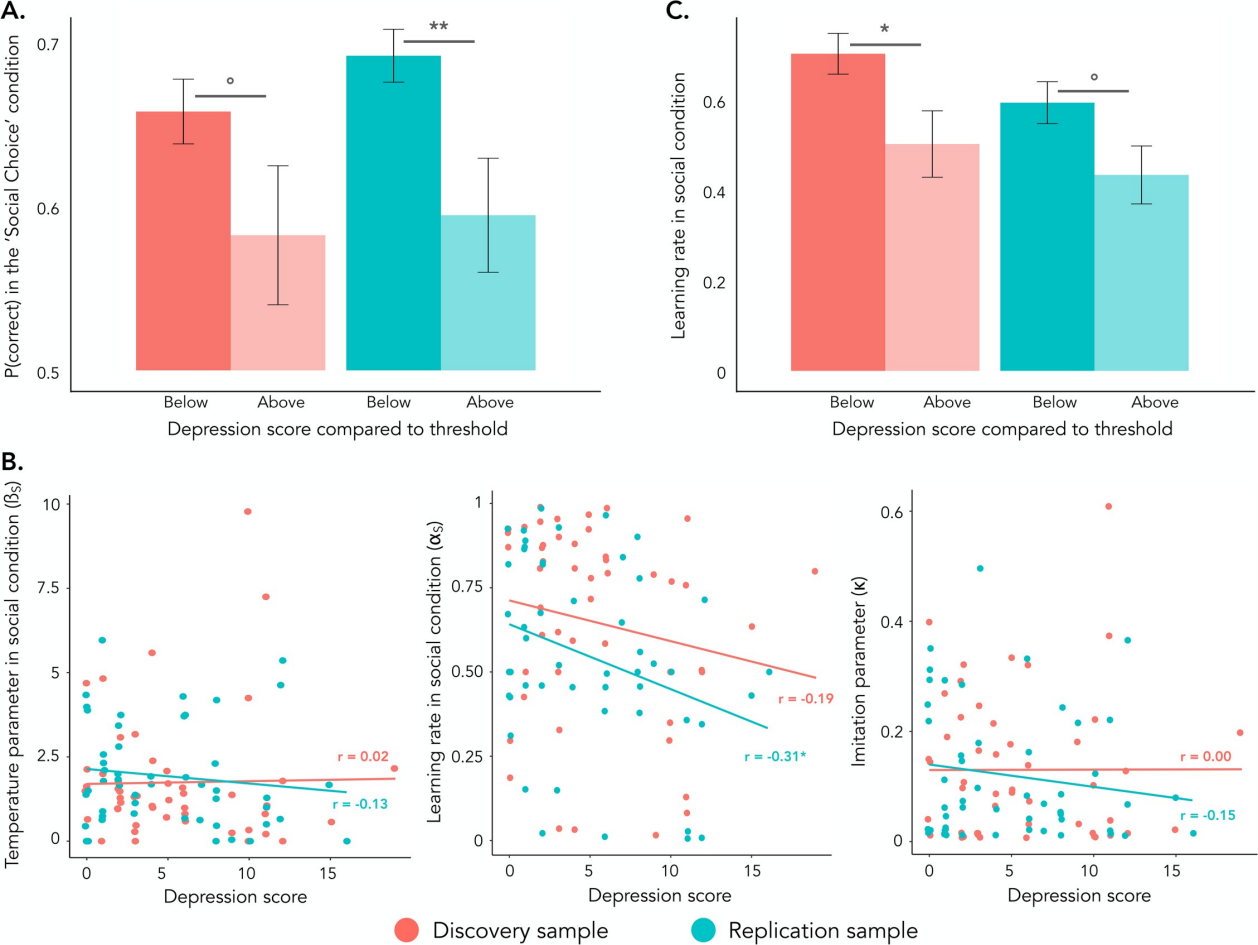
**Classification and computational results (A) Model-free classification.** The correct choice rate difference between the ‘Private’ and the ‘Social-Choice’ conditions was significantly different between participants with ‘Absent’ and ‘Present’ depressive symptoms. **(B) Effect of depression scores on the learning rate in the social context**. Higher depression scores were associated with lowered learning rates in the social contexts but not with a significant effect on the two other parameters fitted on the ‘Social-Choice’ condition **(C) Model-based classification.** The difference between the learning rate of the ‘Private’ and the social information contexts was significantly different between participants with ‘Absent’ and ‘Present’ symptoms of depression. Present symptoms of depression correspond to scores ≥ 8 on the HAD depression subscale, respectively. Error bars represents standard errors to the mean. ‘r’ = Pearson’s correlation coefficient., °p<0.10, *p<0.05, Student’s t-test and Pearson’s correlation.

Although model-free analyses reveal a robust negative effect of depressive symptoms on learning in the ‘Social-Choice’ condition, they do not elucidate the cognitive mechanisms underlying this effect. Indeed, the effect of depressive symptoms could either be due to differences in social information processing, such as the demonstrator’s choices and outcomes (i.e. a *primary* social learning deficit) or to differences in the weighting of the information generated by participants’ own choices when social information is also available (i.e. a *secondary* social learning deficit or *audience effect*). These two hypotheses are hard to tease apart based on raw behavioral analyses, because both predict a reduced correct choice rate in the ‘Social’ conditions. Thus, to arbitrate between these two possibilities, we fitted a previously validated social reinforcement learning model [*14*, *24*]. This model allows for biasing participants’ choice depending on the demonstrator’s choice in the ‘Social-Choice’ condition (i.e. *imitation*) and to update the value attributed to each symbol depending on the demonstrator’s outcome in the ‘Social-Choice+Outcome’ condition (i.e. *vicarious trial-and-error*). To directly assess the ‘socially induced individual learning deficit’ hypothesis [*14*], we allowed participants to have different individual learning parameters in the ‘Private’ (learning rate: *α*_*P*_ ,temperature parameter: *β*_*P*_) and in the two social conditions (‘Social-Choice’ and ‘Social-Choice+Outcome’ conditions: *α*_*S*_ , *β*_*S*_; **Fig 5A**).

More precisely, individual learning and decision-making were modeled with classical softmax (Eq 1) and delta-rule (Eq 2) functions, respectively governed by learning rate and choice randomness (or temperature) parameters:

$$P_{t}({s_{t},a}_{t})=1/{(1+e}^{(\Delta Q_{t}(s_{t}))*\beta })$$ (1)

$$Q_{t+1}(s_{t},a_{t})=Q_{t}(s_{t},a_{t})+\alpha_{P}*{RPE}_{t}$$ (2)

Where RPEt is the reward prediction error calculated as follows (Eq 3):

$${RPE}_{t}=R_{t}-Q_{t}(s_{t},a_{t})$$ (3)

During the ‘Social-Choice’ condition, the model assumes that the Demonstrator’s choice induces an ‘action’ prediction error (*APE*_*t*_; (Eq 4)), which measures how surprising the Demonstrator’s choice is, given the subject’s current estimate of the probability of selecting this option:

$${APE}_{t}=1-P_{t}(s_{t},a_{t})$$ (4)

The APEt is then used to bias choice probability (Eq 5) in the subsequent trial and the effect is scaled by a parameter *κ* ∈ {0–1}:

$$P_{t+1}(s_{t},a_{t})=P_{t}(s_{t},a_{t})+\kappa *{APE}_{t}$$ (5)

Finally, in the ‘Social-Choice+Outcome’ trials, the model assumes that the demonstrator’s outcome induces an ‘observational’ reward prediction error (Eq 6), which is scaled by observational learning rate *α*_*O*_ ∈ {0–1} (Eq 7):

$${OPE}_{t}={R(demonstrator)}_{t}-Q_{t}(s_{t},a_{t})$$ (6)

$$Q_{t+1}(s_{t},a_{t})=Q_{t}(s_{t},a_{t})+\alpha_{O}*{OPE}_{t}$$ (7)

To sum up, this computational model allowed us to address both *primary* social learning deficits (*i*.*e*. learning deficits captured by the parameters *κ* and *α*_*O*_, which are specific to social information) and *secondary* social learning deficits (*i*.*e*. learning deficits captured by the parameters *β*_*S*_ and *α*_*S*_, which are specific to individual learning in contexts where social information is available).

### Computational effects of depressive symptoms

As previously, we analyzed the model parameters fitted on participants’ actual behavior using correlations. Higher depression scores were specifically associated with lower learning rates in the ‘Social’ conditions (*r*_*META*_ = -.25 ± 0.10, *z*_*META*_ = -2.55, *p* = .011; all others, including anxiety: |*z*_*META*_| < 1.30, all *p*s > .190; **Fig 5B–5D**). These results where further confirmed by with structural equation modeling accounting for the correlation between the parameters (depression scores: *z*_*META*_ = -2.61, *p* = .009; other *p*s > .188; **Fig 4C**). Interestingly, high depression scores were not solely associated with decreased learning rates in the ‘Social’ conditions, but also with decreased learning rates in the ‘Social’ conditions when controlling for the learning rates in the ‘Private’ condition (*z*_*META*_ = -3.08, *p* = .002), which indicates that the presence of social information decreased the learning rate of the most depressed participants. To assess the complementary utility of computational measures, we tested whether the learning rate in the ‘Social’ conditions could identify participants with symptoms of depression (i.e. HAD depression subscale score equal or above 8 [*21*]). The difference in learning rates detected participants with depressive symptoms (score ≥ 8) with good accuracy (64 ± 1%), good sensitivity (64 ± 2%) and good specificity (65 ± 3%). A comparison between a classifier based on the model parameters and a classifier based on correct choice rates revealed that the model-based classifier was more specific to detect participants with higher symptoms of depression (*t*(198) = 5.86, *p* < .001), but was less sensitive (*t*(198) = -12.03, *p* < .001; **Fig 4C**) than the classifier based on correct choice rates.

### Model simulations analyses

Model-based analyses indicated that the severity of depressive symptoms specifically reduced individuals’ learning rate in ‘Social’ conditions (*α*_*S*_): a parameter that is used both in the ‘Social-Choice’ and in the ‘Social-Choice+Outcome’ condition. Model-free behavioral analyses showed that the learning deficit associated with depressive symptoms was specific to the ‘Social-Choice’ condition. To ascertain that this computational result was compatible with our model-free observation, we ran the same statistical analysis on simulated data [25]. Crucially, data simulated using the fitted parameters accurately recovered the decrease in performance associated with depression scores in the ‘Social-Choice’ condition compared to the ‘Private’ condition using the same mixed linear regression as on behavioral data (*z*_*META*_ = -2.72, *p* = .007) as well as the blunted effect of depression scores in the ‘Social-Choice+Outcome’ condition compared to the ‘Private’ condition (*z*_*META*_ = -1.74, *p* = .082). Therefore, it appears that, although depressive symptoms are associated with decreased learning rates in both social conditions, its detrimental effect is manifest only in the ‘Social-Choice’ condition. This is probably due to showing the demonstrator’s outcomes in the ‘Social-Choice+Outcome’ condition. This additional outcome information may compensate for the decreases learning rates with depressive symptoms. Confirming this intuition, our simulation analyses accurately recovered the absence of significant effect of depressive symptoms in the ‘Private’ condition (*z*_*META*_ = -0.29, *p* > .250; **S6 Fig**). Thus, the simulations captured the specificity of the behavioral effect of depression scores and illustrate that our model provides an accurate description of the data.

### Checking parameter recovery

As we were interested in the modulation of specific parameters by depression scores we tested whether our task allowed us to successfully retrieve a correlation between parameters in simulated datasets, an important quality check often referred to as ‘parameter recovery’ [*25*]. To do so, we ran 100 sets of simulations for each parameter, each simulating 100 participants, with the parameter of interest correlating with an arbitrary variable (defined as the depression scores) and the other parameters being randomly set for each participant in the range obtained by optimization on the total sample. The simulated data were then fitted using our social reinforcement-learning model. Overall parameter recovery was very good, especially for the parameters of the social conditions, with significant correlations were found in the 100% of the simulated datasets (average correlation coefficient of the parameters: *r* = 0.73 ± 0.01). Importantly, the recovery of the correlations was specific to the manipulated parameter with false alarms detected in less than 10% of the cases except for learning rate and choice temperature in the ‘Private’ condition (which was not our condition of interest) (**Fig 5B**). This result indicates that it is very unlikely that a correlation of one of our parameters with participants’ HAD depression scores is due to an effect of depression scores on another parameter.

## Discussion

In the present study we assessed reinforcement learning with a behavioral paradigm involving both private and social contexts, while concomitantly assessing depressive and anxiety symptoms in the general population. First, we replicate previous findings showing that participants integrate the demonstrator’s choices and outcomes, which is consistent with the idea that social learning processes (both in terms of imitation and vicarious trial-and-error) play a role in human reinforcement learning [*14*, *15*, *26*–*28*]. Second, we show that the severity of depressive symptoms is associated with a learning impairment that is specific to the learning context where participants are informed about the demonstrator’s choices (social context). This negative effect was robust to the inclusion of anxiety, and robust across experiments and outcome contingencies. Finally, computational analyses allowed us to characterize the effect of depressive symptoms as a secondary social learning deficit, i.e. a reduction of the learning rate in social contexts.

We found that depressive symptoms had a specific effect on imitation in the ‘Social-Choice’ condition. Crucially, the effect was robust to the inclusion of anxiety, which did not modulate performance in our task. That anxiety had no effect may come as a surprise given that previous studies have found that anxiety is associated with deficits in social and non-social reinforcement learning [*29*]. One possible explanation is that anxiety might be more strongly linked to classical fear conditioning than reward-based instrumental learning [*30*]. Depressive symptoms might thus undermine social reinforcement learning in instrumental and reward-maximization contexts, while anxiety might affect the same processes when outcomes are independent from the participants’ choices (i.e. Pavlovian learning) and when outcomes have a negative valence (aversive contexts).

Model-free analyses *per se* do not allow us to pinpoint the psychological mechanisms underlying the negative effect of depressive scores on correct choice rates in the ‘Social-Choice’ context. The absence of interaction between the demonstrator’s performance and depressive symptoms suggests that depressive symptoms did not lead participants to disproportionally follow ‘bad examples’ or to be insensitive to ‘good examples’. However, interpretations based on negative results are, at best, unsafe. To formally characterize the psychological mechanisms of the detrimental effects of depressive symptoms we thus turned to model-based analyses.

We fitted subjects’ choice with a slightly modified version of a previously validated social reinforcement-learning model [*14*]. As in standard algorithms, the model assumes that subjects learn option values via the calculation of a reward prediction error, that the values are moderated by a learning rate (α_*P*_) and that choices are generated via a soft-maximization process whose stochasticity is governed by a temperature (β_*P*_) [*31*]. In addition to this ‘private’ learning module, the model also displays sensitivity to social information: in the ‘Social-Choice’ condition the demonstrator’s choice biases the subsequent subject’s choice (the magnitude of this effect is governed by an imitation rate κ) and in the ‘Social-Choice+Outcome’ condition the demonstrator’s outcome is integrated into the subject’s value function with a vicarious learning rate (**α**_*O*_). Finally, we also allowed for different private learning rates and temperatures in the ‘Social’ contexts (α_*S*_ and β_*S*_). This precise model parameterization allowed us to disentangle two different hypotheses concerning the drop in performance associated with depressive symptoms in the ‘Social-Choice’ condition. A correlation between depressive scores and imitation rates and/or vicarious learning rates would imply what we define a ‘primary’ social learning impairment (i.e. an impairment of the social learning processes *per se*). On the contrary, a correlation between the ‘Social’ context-specific learning rate and/or temperature would imply a ‘secondary’ social learning impairment (i.e. an impairment of the private learning processes in presence of social information). We found that depressive scores negatively correlated with the private learning rate in the social context (**α**_*S*_), thus indicating that the effect was consistent with a secondary impairment and was specific to the learning (as opposed to the decision) process. In other words, our computational results suggest that one possible way in which depressive symptoms affect learning in social contexts is conceptually similar to a negative audience effect [*32*, *33*], where the presence of social signals (the demonstrator’s choices) induces a reduction of subjects’ instrumental performance.

From a methodological point of view, our study exemplifies how computational approaches can provide new insights on the way in which cognitive processes vary with clinical symptoms. Indeed, computational modeling demonstrated that the effect of depressive symptoms was selective of the way individual information was processed [*34*, *35*]. It is worth noting that these conclusions were only allowed after a careful testing of the ability of our task to precisely identify which model parameter was influenced by depressive symptoms [*25*]. The exact cognitive and psychological mechanisms that mediate the negative effect of social signals in instrumental performance remain to be characterized. One possibility given that depressive symptoms are associated with lower cognitive functioning in general [*36*] is that the mere presence of others exacerbates these difficulties by capturing already scarcer attentional resources. Alternatively, negative perception of self and negative comparison to others are core symptoms of depressive symptoms [*37*]. Therefore, it is possible that the most depressed participants perceived their demonstrator’s behavior as more reliable, thus underweighting the information they acquired through their own experience.

Our results provide new evidence that depression-related reward learning deficits are highly context-dependent [*3*–*5*], and suggest that the difference in learning rates associated with depressive symptoms may only arise in social contexts [*5*, *9*]. Crucially, our results suggest that supposedly neutral aspects of the experimental setup (such as whether or not the task is done in the presence or absence of an experimenter), may affect the results and explain inconsistent findings [*38*]. In line with recent propositions, our results also suggest that a deeper investigation of socio-cognitive impairments in depressive symptoms may provide important new insights [*10*, *11*]. Following this idea, it would be particularly interesting to contrast the effect of depressive symptoms on learning when the information is socially (as in the current study) compared to asocially provided. Finally, we suggest that developing tools assessing reward learning outside and inside social contexts (characterized either by the presence of another player or by the social nature of the outcomes [*39*]) may prove useful to improve diagnosis and personalize treatments of depressive syndromes in the long term.

An obvious limitation of our study, is that we did not control for participants’ actual diagnosis and treatment, which may be problematic since medication interacts with decision-making in depression [*40*]. Therefore, our results would benefit from being replicated in carefully characterized population, while controlling for medication status and medical history. This replication would allow us to further measure the diagnostic value of our behavioral task and associated computational model-based analyses. Indeed, in the present study, we only tested its ability to detect participants with depressive symptoms as identified by a self-rated scale [*21*] . It would be particularly interesting to test whether our behavioral and computational measures improve existing self-assessments that detect clinically diagnosed cases of depression [*41*]. Finally, longitudinal designs will be required to assess whether or not our behavioral and computational measures present good test-retest reliability and reflect states or traits, and whether or not they predict the evolution of depressive symptoms to clinical diagnosis.

Our results have implications beyond their clinical relevance. Consistent with the ‘social learning theory’ participants imitated demonstrators’ choices (‘Social-Choice’ condition) and learned from their outcomes (‘Social-Choice+Outcome’ condition) [*13*, *14*]. At the behavioral level, these two psychological processes were manifest in the fact that participants’ performance was modulated by the demonstrators’ performance. In particular, we found that participants observing a demonstrator performing ‘well’ performed better in the social compared to the private learning context. Importantly, the opposite was also true: participants observing low performing demonstrators displayed lower performance in the social compared to the private context. This latter result is in apparent contrast with the normative view that imitation should be biased toward successful individuals in order to be evolutionary adaptive [*42*–*44*]. This is also in contrast with recent empirical evidence using a very similar paradigm and showing that imitation rate is modulated by the actual performance of the demonstrator, so that demonstrators making random (i.e., non reward-maximizing) decisions are less imitated [*15*]. Two differences between the previous design and ours may explain this discrepancy. First, the previous study involved mild electric shocks (primary reinforcer), while our study involved abstract points to be converted into money (secondary reinforcer). More importantly perhaps, the previous design involved a between-subjects design with two groups of participants paired either with a consistently good or with a consistently bad participant, while in our experiments the performance of the demonstrator was allowed to fluctuate in a within-subject manner around an optimal behavior. Therefore, it could also be argued that our experiment is not well-suited for measuring demonstrators’ performance effects on participants’ imitation behavior as such effects require a relatively long and stable reputation building process [*45*, *46*].

The question remains whether or not social learning in our task (imitation and vicarious trial-and-error) engaged domain-specific social cognitive module or domain-general information processing modules. In the absence of additional data (such as neuroimaging) we cannot provide a definitive answer. However, evidence from post-learning face ratings provides some clues [*47*]. We found a positive correlation between performance in the social contexts and the demonstrator’s judgment of trustworthiness. Even if we cannot infer a causal link and its direction from the post-learning face evaluation, these results suggest that a specific socio-cognitive module (face evaluation) correlated with instrumental performance, thus demonstrating the engagement of social information-specific processing and our reinforcement learning task.

## Materials and methods

### Participants

Two independent cohorts of 100 American participants, similar in terms of reported age (mean reported age across the two cohorts: 33.39 ± 2.03) and of reported male/female ratio (mean reported male/female ratio across the two cohorts: 35%; see Table 1) were recruited via Amazon Mechanical Turk to participate in this online study. Each participant received a fixed 4$ amount for completing the 40-minute task to which a bonus earned during the experiment was then added (average bonus: 0.49$). Participant received a description of the study and signed an informed consent before starting the experiment. The study was approved by the the local Ethical Committee (Conseil d’évaluation éthique pour les recherches en santé–CERES n°201659) and is in accordance with the Declaration of Helsinki (World Medical Association, 2008). The first cohort corresponded to a ‘discovery experiment’ where we explored the relation between instrumental performance and clinical scores; the second cohort corresponded to a ‘replication experiment’ where we tested the robustness and replicability of the effect identified in the first experiment.

### Experimental design

Participants performed the probabilistic instrumental learning task described in the Results section (**Fig 1A and 1B**). The task was programmed on Qualtrics and was composed of six learning blocks of 20 trials each. In each block, participants had to choose between two cues. Cues were characters of the agathodaimon font and were always presented in pair and only in one block *per* subject. The cue-to-condition attribution was randomized across subjects. Participants made their choice by pressing the E or P keys to choose the leftmost or rightmost symbol. Participants were given no explicit information on reward probabilities, which they had to learn through trial and error. In addition, they were encouraged to accumulate as many points as possible, with their final amount of points being translated into bonus money at the end of the experiment (conversion rate: 40 points equals 1$ bonus). In each pair, cues were associated with reciprocal reward probabilities (20/80% or 30/70%). For instance, in a 30/70% pair, the most rewarded cue provided a positive outcome (+1 point) 70% of the times and a negative outcome (-1 point) 30% of the time, while the less rewarded cue provided a negative outcome 70% of the time and a positive outcome 30% of the time. Participants had unlimited time to make their choice (Mean reaction time: 2.47 ± 0.88 s, no significant effect of depressive symptoms were found on the reaction times, all *p*s > .250).

Participants were told they had been paired with another player at the beginning of the experiment with whom they played in turn in each trial. In addition, it was indicated that there was no competition between them and the other player and that each player played for her/himself. As in previous studies [48], the behavior of the demonstrators was determined by a reinforcement learning algorithm (Q-learning) with a reasonable set of free parameters (𝛼 = 0.5, *ß* = 10; see below for a description for the Q-learning and its parameters). To avoid social perceptual biases, the other player was represented by a neutral avatar, chosen to be generally perceived as neither dominant or submissive nor trustworthy or untrustworthy [*49*]. Participants had to choose their own avatars in a set of other 16 identities (8 female, 8 male) at the beginning of the task. Participants performed this task in three different contexts with different amounts of social information: a ‘Private’ condition in which they did not have access to the demonstrator’s behavior, a ‘Social-Choice’ condition in which participants could see the demonstrator’s behavior but not their outcomes and a ‘Social-Choice+Observation’ in which participants could observe the demonstrator’s decisions and outcomes. Importantly, participants performed each condition (‘Private’, ‘Social-Choice’ and ‘Social-Choice+Outcome’) in separate blocks and each block was repeated twice. In the ‘Stable’ type of contingency, outcome probabilities were set at 30/70% and did not change during the block. In the ‘Reversal’ type of contingency, outcome probabilities were set at 20/80% and was inverted across cue after 10 trials (in average). Finally, at the end of the experiment, participants rated their demonstrator’s avatar on three personality traits (trustworthiness, dominance and competence) and completed the Hospital Anxiety and Depression Scale [*21*] as well as the Peters et al. Delusions Inventory, that was included in the exploratory analysis of the Discovery sample and then discarded in absence of any significant effect and its inclusion did not affect the effect of depression. The total procedure lasts approximatively 45 minutes.

### Statistical analyses

The analyses were performed on all participants and trials. No exclusion criteria was applied.

#### Percentage of correct choices

Percentage of correct choices were extracted for each block and either correlated or used as a continuous dependent variable.

#### Meta-analysis

Meta-analyses were ran using a mixed-effects model which is a conservative method for computing meta-analytic effects across studies. More precisely, this method weights each study depending on its variability and allows non-random differences in effect sizes between samples and computes the average of the distribution of the effect sizes. These analyses were performed using R Metafor package [*50*].

#### Regression analyses

A mixed linear regression with both random intercept and random slopes was conducted on correct choice rates taking participants’ ID as a random factor, condition (‘Private’, ‘Social-Choice vs ‘Social-Choice+Outcome’) as within-subject variables and depression and anxiety scores as well as demonstrator’s performance and trustworthiness judgment as continuous between-subject variables (**Table 3**).

#### Classification analyses

Out of sample tests were used to assess whether our task was able to distinguish participants scoring above the ‘depressive symptoms absent’ threshold in depression scale from those below this threshold. 50 participants were randomly extracted from the entire sample and used to optimize a classifier of depressive symptoms (HAD depression subscale score above or equal to 8 [*21*]) using either the correct choice rates in the ‘Social-Choice’ condition (model-free measure) or the learning rates in the Social information conditions (*α*_*S*_ model-based measure; see below). The optimal cut-off was defined to jointly maximize the specificity (true negative rate) and the sensitivity (true positive rate) of the classifier on the training sample. The classifier and the associated optimal cut-off was tested on the 50 remaining participants. This operation was repeated 100 times in order to estimate the average accuracy, sensitivity and sensibility of the classifiers.

### Computational analyses

#### Model fitting

Computational analyses were performed after the collection of the replication sample. However, in order to assess the robustness of our computational model, our computational results are presented as a meta-analysis across the exploratory and replication samples (**S2 Table**).

We optimized the model parameters by minimizing the Laplace approximation to the model evidence (log of the posterior probability: LPP) (Eq 8):

$$LPP=log(P(\mathrm{data}|\theta_{1,\ldots n}))+\sum_{k=1}^{n}log(P(\theta_{k}))$$ (8)

Where D represents the data, *θ*_1,…*n*_ the model, and *θ*_*k*_ represents one of the *n* parameters of the computational model. The LPP represents a trade-off between the model’s accuracy and complexity: it increases with the likelihood of the model given the data (a measure of fit) and decreases with the number of parameters. By including priors over the parameters, this method avoids degenerate parameter estimation. In our analysis, the priors were defined as a gamma function (gampdf(1.2,5)) for the temperature parameters (range: 0<β<Infinite) and as a beta function (betapdf(1.1,1.1)) for the learning and imitation rates (ranges: 0<α<1, 0<κ<1) as described in [*51*] (see **Table 4** for the estimated parameters).

**Table 4:** Estimated model parameters for the actual participants and for the simulated virtual demonstrators (mean ± 95% c.i.).

	Participants	Virtual demonstrators
β_P_	2.20 ± 0.47	9.54 ± 0.49 (real: 10)
α_P_	0.58 ± 0.05	0.52 ± 0.02 (real: 0.50)
β_S_	1.83 ± 0.34	
α_S_	0.60 ± 0.06	
κ	0.13 ± 0.02	
α_O_	0.46 ± 0.06	

Importantly, LPP analysis suggested that the social reinforcement learning fit the data better than a simple Q-learning model without social influence, even accounting for its extra-complexity (social reinforcement learning model: posterior probability: 90 ± 3%; exceedance probability: 100%). As a control analysis, in order to ensure that our model comparison criterion was not over-fitting prone, we fit the behavior of the virtual demonstrators that we generated with a Q-learning model. This model recovery analysis [*25*] correctly indicated that the simple Q-learning model explained the demonstrators’ data better (social reinforcement learning model: posterior probability: 100 ± 0%; exceedance probability: 100%) (see supplementary figures and table for additional information concerning the parameter recovery analysis).

Because the model parameters were correlated with each other (maximal correlation: *r* = 0.53; **S4 Table**), we used structural equation modeling in addition to correlation analyses to analyze the influence of depression scores on the model parameters. This technique allowed us to test the influence of depression scores on each parameter while simultaneously accounting for the inter-correlations of the dependent variables (the model free parameters) and of the independent variable (the depression score).

#### Model simulation analyses

Finally, we assessed the ability of the model to recover the observed behavioral effect of depressive symptoms using model simulations [*25*]. For each participant, we simulated behavioral data for each condition based on their best fitting parameters. Importantly, a simulated demonstrator was also generated, such that the simulated data were completely independent of the contingencies actually experienced by the participants. This procedure was repeated 100 times, to avoid any effect of participant’s and demonstrator’s history of choice and outcomes. The analysis of the recovered percentage of correct choices was ran on the averaged rates of correct choices across the 100 simulations using a linear mixed regression taking the exact same predictors as the mixed general linear model used for analyzing participants’ percentage of correct choices.

## Supporting information

S1 Table Effect of the depression scores on the probability of choosing the most rewarded symbol in the two samples computed by the mixed linear regression. (DOCX)

S2 Table Effect of depression scores on each model parameter in the two samples (obtained by structural equation modelling; ± represents s.e.m.). (DOCX)

S3 Table Effect of depression scores on the simulated probabilities of choosing the most rewarded symbol in each sample (mixed linear regression). (DOCX)

S4 Table Correlation matrix between the model parameters. (DOCX)

S5 Table Meta-analytic model with reversal as cofactor. (DOCX)

S6 Table Parameter recovery—Correlation between the recovered parameters and the Depressive symptoms scores for each parameter manipulation. (DOCX)

S1 Fig Distribution of Depression scores in the two samples. (PDF)

S2 Fig Effect of depression scores on the correct response rate for each sample and each reward contingency. (PDF)

S3 Fig Learning curves in each condition for each reward contingency. Mean learning curves (in black) and their standard errors (shaded light grey area) are represented for each condition and each reward contingency. The dotted line in orange represents the model prediction for each condition and each reward contingency. The grey area for the reversal blocks indicates the trials in each the reversal of reward contingencies can occur. For each plot, the top dotted line indicates the matching law and the bottom dotted line indicates chance level.
(PDF)

S4 Fig Correlation between the actual performances and the performances predicted by the model. For each of the condition, the performance predicted by the computational model highly correlated with the participants’ actual performances in both the discovery and the replication samples (Meta-analytic correlations: all *r*-s > .72, all *z*-s > 10.21, all *p*-s < .001).
(PDF)

S5 Fig Distribution of the model parameters in the two samples. (PDF)

S6 Fig Effect of depressive symptoms on the rate of correct choice in the social contexts. Depressive symptoms (HAD Depression subscale score ≥ 8) were associated with decreased correct response rate only in the ‘Social Choice’ condition. This effect was accurately recovered by simulations of our model (white dots). Error bars represent standard errors.
(PDF)

S7 Fig Correlation between the regression coefficients in the discovery and replication samples. The correlation coefficients of the two samples were highly correlated, indicating the replication of the results in the two samples. The dotted line corresponds to the perfect replication.
(PDF)

S8 Fig Model comparison between a private learning model, our social learning model and a model with three learning rates and three temperature parameters. In order to further test the robustness of our results we first compared our model with a more complex model including different learning rates and temperature parameters for each condition. This parsimony-driven model comparison including this model confirmed that the one we used in our analyses better accounted our data. We then compared our model with all the models of the possible models containing one to three learning rates and one to three temperature parameters or two temperature parameters in addition to the imitation (κ) and the observation learning rate parameter (α_O_) and a simple reinforcement learning model. In line with our results, the model with two learning parameters and one temperature parameter was the most probable for our data (S9 Fig). In addition, we recovered the specific association between higher depression scores and lower learning rates in the social conditions with the learning parameters estimated in this model (b = -0.2 ± 0.01, z = -2.55, p = .011, all other |z| < 1.48, all p-s > .137; S10 Fig).
(PDF)

S9 Fig Model comparison between learning models of increasing complexity. (PDF)

S10 Fig Correlation between learning rates in the Social Contexts (retrieved from a model with only one temperature) and Depression score. The correlation is also significant (*b* = -0.2 ± 0.01, *z* = -2.55, *p* = .011).
(PDF)

## References

[1] Pizzagalli D. A., Jahn A. L., O’Shea J. P., Toward an objective characterization of an anhedonic phenotype: A signal-detection approach. *Biological Psychiatry*. 57, 319–327 (2005). 10.1016/j.biopsych.2004.11.026 15705346 PMC2447922

[2] Kennedy S. H., Core symptoms of major depressive disorder: relevance to diagnosis and treatment. *Dialogues Clin Neurosci*. 10, 271–277 (2008). 18979940 10.31887/DCNS.2008.10.3/shkennedy PMC3181882

[3] Chen C., Takahashi T., Nakagawa S., Inoue T., Kusumi I., Reinforcement learning in depression: A review of computational research. *Neuroscience &* *Biobehavioral Reviews*. 55, 247–267 (2015). 10.1016/j.neubiorev.2015.05.005 25979140

[4] Eshel N., Roiser J. P., Reward and Punishment Processing in Depression. *Biological Psychiatry*. 68, 118–124 (2010). 10.1016/j.biopsych.2010.01.027 20303067

[5] Huys Q. J., Pizzagalli D. A., Bogdan R., Dayan P., Mapping anhedonia onto reinforcement learning: a behavioural meta-analysis. *Biol Mood Anxiety Disord*. 3, 12 (2013). 10.1186/2045-5380-3-12 23782813 PMC3701611

[6] Hägele C. et al, Dimensional psychiatry: reward dysfunction and depressive mood across psychiatric disorders. *Psychopharmacology*. 232, 331–341 (2015). 10.1007/s00213-014-3662-7 24973896 PMC4297301

[7] Rothkirch M., Tonn J., Köhler S., Sterzer P., Neural mechanisms of reinforcement learning in unmedicated patients with major depressive disorder. *Brain*. 140, 1147–1157 (2017). 10.1093/brain/awx025 28334960

[8] Rutledge R. B. et al, Association of Neural and Emotional Impacts of Reward Prediction Errors With Major Depression. *JAMA Psychiatry*. 74, 790–797 (2017). 10.1001/jamapsychiatry.2017.1713 28678984 PMC5710549

[9] Chung D. et al, Valuation in major depression is intact and stable in a non-learning environment. *Sci Rep*. 7 (2017), 10.1038/srep44374 28281665 PMC5345037

[10] Kupferberg A., Bicks L., Hasler G., Social functioning in major depressive disorder. *Neuroscience & Biobehavioral Reviews*. 69, 313–332 (2016). 27395342 10.1016/j.neubiorev.2016.07.002

[11] Weightman M. J., Air T. M., Baune B. T., A Review of the Role of Social Cognition in Major Depressive Disorder. *Front*. Psychiatry. 5 (2014), 10.3389/fpsyt.2014.00179 25566100 PMC4263091

[12] Fussner L. M., Mancini K. J., Luebbe A. M., Depression and Approach Motivation: Differential Relations to Monetary, Social, and Food Reward. *J Psychopathol Behav Assess*, 1–13 (2017). 10.1007/s10862-016-9556-8 28286370 PMC5342840

[13] Bandura A., Social learning theory. *Morristown* (NJ: General Learning Press, 1971).

[14] Burke C. J., Tobler P. N., Baddeley M., Schultz W., Neural mechanisms of observational learning. *PNAS*. 107, 14431–14436 (2010). 10.1073/pnas.1003111107 20660717 PMC2922583

[15] Selbing I., Lindström B., Olsson A., Demonstrator skill modulates observational aversive learning. *Cognition*. 133, 128–139 (2014). 10.1016/j.cognition.2014.06.010 25016187

[16] Medin D., Ojalehto B., Marin A., Bang M., Systems of (non-)diversity. *Nature Human Behaviour*. 1, 0088 (2017).

[17] Gillan C. M., Kosinski M., Whelan R., Phelps E. A., Daw N. D., Characterizing a psychiatric symptom dimension related to deficits in goal-directed control. *eLife Sciences*. 5, e11305 (2016). 10.7554/eLife.11305 PMC4786435 26928075

[18] Gillan C. M., Daw N. D., Taking Psychiatry Research Online. *Neuron*. 91, 19–23 (2016). 10.1016/j.neuron.2016.06.002 27387647

[19] Shapiro D. N., Chandler J., Mueller P. A., Using Mechanical Turk to Study Clinical Populations. *Clinical Psychological Science*. 1, 213–220 (2013).

[20] Collaboration O. S., Estimating the reproducibility of psychological science. *Science*. 349, aac4716 (2015). 10.1126/science.aac4716 26315443

[21] Zigmond A. S., Snaith R. P., The Hospital Anxiety and Depression Scale. *Acta Psychiatrica Scandinavica*. 67, 361–370 (1983). 6880820 10.1111/j.1600-0447.1983.tb09716.x

[22] Wigman J. T. W. et al, Evidence That Psychotic Symptoms Are Prevalent in Disorders of Anxiety and Depression, Impacting on Illness Onset, Risk, and Severity—Implications for Diagnosis and Ultra–High Risk Research. *Schizophr Bull*. 38, 247–257 (2012). 10.1093/schbul/sbr196 22258882 PMC3283146

[23] Regier D. A., Rae D. S., Narrow W. E., Kaelber C. T., Schatzberg A. F., Prevalence of anxiety disorders and their comorbidity with mood and addictive disorders. *The British Journal of Psychiatry*. 173, 24–28 (1998). 9829013

[24] Puskaric M., von Helversen B., Rieskamp J., How social and non-social information influence classification decisions: A computational modelling approach. *The Quarterly Journal of Experimental Psychology*. 70, 1516–1534 (2017). 10.1080/17470218.2016.1192209 27311016

[25] Palminteri S., Wyart V., Koechlin E., The Importance of Falsification in Computational Cognitive Modeling. *Trends in Cognitive Sciences*. 21, 425–433 (2017). 10.1016/j.tics.2017.03.011 28476348

[26] Biele G., Rieskamp J., Krugel L. K., Heekeren H. R., The Neural Basis of Following Advice. *PLoS Biol*. 9, e1001089 (2011). 10.1371/journal.pbio.1001089 21713027 PMC3119653

[27] Selbing I., Olsson A., Beliefs about Others’ Abilities Alter Learning from Observation. *Scientific Reports*. 7, 16173 (2017). 10.1038/s41598-017-16307-3 29170461 PMC5701038

[28] Vostroknutov A., Polonio L., Coricelli G., The Role of Intelligence in Social Learning. *Scientific Reports*. 8, 6896 (2018). 10.1038/s41598-018-25289-9 29720699 PMC5932062

[29] Bach D. R., Dolan R. J., Knowing how much you don’t know: a neural organization of uncertainty estimates. *Nature Reviews Neuroscience*. 13, 572–586 (2012). 10.1038/nrn3289 22781958

[30] Olsson A., Phelps E. A., Social learning of fear. *Nature Neuroscience*. 10, 1095–1102 (2007). 10.1038/nn1968 17726475

[31] Watkins C. J. C. H., Dayan P., Q-learning. *Mach Learn*. 8, 279–292 (1992).

[32] Zajonc R. B., Social Facilitation. *Science*. 149, 269–274 (1965). 10.1126/science.149.3681.269 14300526

[33] Hazem N., George N., Baltazar M., Conty L., I know you can see me: Social attention influences bodily self-awareness. *Biological Psychology*. 124, 21–29 (2017). 10.1016/j.biopsycho.2017.01.007 28111232

[34] Huys Q. J. M., Maia T. V., Frank M. J., Computational psychiatry as a bridge from neuroscience to clinical applications. *Nature Neuroscience*. 19, 404–413 (2016). 10.1038/nn.4238 26906507 PMC5443409

[35] Montague P. R., Dolan R. J., Friston K. J., Dayan P., Computational psychiatry. *Trends in Cognitive Sciences*. 16, 72–80 (2012). 10.1016/j.tics.2011.11.018 22177032 PMC3556822

[36] Rock P. L., Roiser J. P., Riedel W. J., Blackwell A. D., Cognitive impairment in depression: a systematic review and meta-analysis. *Psychological Medicine*. 44, 2029–2040 (2014). 10.1017/S0033291713002535 24168753

[37] Alloy L. B., Albright J. S., Clements C. M., in Social Processes in Clinical and Counseling Psychology, Maddux J. E., Stoltenberg C. D., Rosenwein R., Eds. (Springer New York, 1987; http://link.springer.com/chapter/10.1007/978-1-4613-8728-2_8), pp. 94–112.

[38] Chevallier C. et al, Susceptibility to the audience effect explains performance gap between children with and without autism in a theory of mind task. *Journal of Experimental Psychology*: *General*. 143, 972–979 (2014). 24392710 10.1037/a0035483 PMC4038654

[39] Chevallier C. et al, Measuring Social Motivation Using Signal Detection and Reward Responsiveness. *PLOS ONE*. 11, e0167024 (2016). 10.1371/journal.pone.0167024 27907025 PMC5132309

[40] Herzallah M. M. et al, Learning from negative feedback in patients with major depressive disorder is attenuated by SSRI antidepressants. *Front*. *Integr*. *Neurosci*. 7 (2013), 10.3389/fnint.2013.00067 24065894 PMC3779792

[41] Fried E. I. et al, Measuring depression over time… Or not? Lack of unidimensionality and longitudinal measurement invariance in four common rating scales of depression. *Psychological Assessment*. 28, 1354–1367 (2016). 10.1037/pas0000275 26821198

[42] Laland K. N., Social learning strategies. *Learn Behav*. 32, 4–14 (2004). 15161136 10.3758/bf03196002

[43] Dall S. R. X., Giraldeau L.-A., Olsson O., McNamara J. M., Stephens D. W., Information and its use by animals in evolutionary ecology. *Trends in Ecology & Evolution*. 20, 187–193 (2005). 16701367 10.1016/j.tree.2005.01.010

[44] Boyd R., Richerson P. J., An evolutionary model of social learning: the effects of spatial and temporal variation. *Social learning: psychological and biological perspectives*, 29–48 (1988).

[45] Ligneul R., Obeso I., Ruff C. C., Dreher J.-C., Dynamical Representation of Dominance Relationships in the Human Rostromedial Prefrontal Cortex. *Current Biology*. 26, 3107–3115 (2016). 10.1016/j.cub.2016.09.015 28094034

[46] Qu C., Ligneul R., Van der Henst J.-B., Dreher J.-C., An Integrative Interdisciplinary Perspective on Social Dominance Hierarchies. *Trends in Cognitive Sciences*. 21, 893–908 (2017). 10.1016/j.tics.2017.08.004 28916140

[47] Oosterhof N. N., Todorov A., The functional basis of face evaluation. *PNAS*. 105, 11087–11092 (2008). 10.1073/pnas.0805664105 18685089 PMC2516255

[48] Suzuki S., Harasawa N., Ueno K., Gardner J. L., Ichinohe N., Haruno M., && Nakahara H. Learning to simulate others' decisions. Neuron, 74(6), 1125–1137 (2012). 10.1016/j.neuron.2012.04.030 22726841

[49] Todorov A., Dotsch R., Porter J. M., Oosterhof N. N., Falvello V. B., Validation of data-driven computational models of social perception of faces. *Emotion*. 13, 724–738 (2013). 10.1037/a0032335 23627724

[50] Viechtbauer W., Conducting meta-analyses in R with the metafor package. *Journal of statistical software*. 36 (2010).

[51] Palminteri S., Khamassi M., Joffily M., Coricelli G., Contextual modulation of value signals in reward and punishment learning. *Nature Communications*. 6, ncomms9096 (2015). 10.1038/ncomms9096 PMC4560823 26302782

