# Supplementary material for: Depressive symptoms are associated with blunted reward learning in social contexts
Source: PLoS Comput Biol. 2019 Jul 29;15(7):e1007224. doi: 10.1371/journal.pcbi.1007224 (PMC6699715; doi:10.1371/journal.pcbi.1007224)
Supplement: S1 Table — (DOCX) [file pcbi.1007224.s001.docx]

**Table S1** - Effect of the depression scores on the probability of choosing the most rewarded symbol in the two samples computed by the mixed linear regression

|  | Discovery sample | Replication sample | Meta-analysis |
| --- | --- | --- | --- |
| Effect of Depressive symptoms in the ‘Private’ condition | *b* = 0.01 ± 0.00,  *t*(46) = 1.57, *p* = .124 | *b* = - 0.00 ± 0.01,  *t*(46) = - 0.58, *p* = .565 | *b* = 0.00 ± 0.01,  *z* = 0.50, *p* = .615 |
| Effect of Depressive symptoms in the ‘Social Choice’ condition vs ‘Private’ condition | *b* = -0.01 ± 0.01,  *t*(239) = -2.66, *p* = .008 | *b* = - 0.01 ± 0.01,  *t*(239) = - 1.17, *p* = .242 | *b* = - 0.01 ± 0.00,  *z* = -2.85, *p* = .004 |
| Effect of Depressive symptoms in the ‘Social Choice+Outcome’ condition vs ‘Private’ condition | *b* = -0.01 ± 0.01,  *t*(239) = -1.21, *p* = .228 | *b* = 0.00 ± 0.01,  *t*(239) = 0.46, *p* = .643 | *b* = - 0.00 ± 0.00,  *z* = -0.83, *p* = .407 |
