# Supplementary material for: Depressive symptoms are associated with blunted reward learning in social contexts
Source: PLoS Comput Biol. 2019 Jul 29;15(7):e1007224. doi: 10.1371/journal.pcbi.1007224 (PMC6699715; doi:10.1371/journal.pcbi.1007224)
Supplement: S2 Table — (DOCX) [file pcbi.1007224.s002.docx]

**Table S2** – Effect of depression scores on each model parameter in the two samples (obtained by structural equation modelling; ± represents s.e.m.)

|  | Discovery sample | Replication sample | Meta-analysis |
| --- | --- | --- | --- |
| ‘Private’ condition temperature parameter (ß_P_) | *M* = 0.03 ± 0.06,  *z* = 0.43, *p* > .250 | *M* = - 0.10 ± 0.09,  *z* = -1.09, *p* > .250 | *M* = -0.02 ± 0.06,  *z* = -0.29, *p* > .250 |
| ‘Private’ condition learning rate (α_P_) | *M* = 0.01 ± 0.01,  *z* = - 0.65, *p* > .250 | *M* = 0.01 ± 0.02,  *z* = 1.14, *p* > .250 | *M* = 0.00 ± 0.01,  *z* = 0.25, *p* > .250 |
| ‘Social’ conditions temperature parameter (ß_S_) | *M* = -0.01 ± 0.02,  *z* = 0.14, *p* > .250 | *M* = - 0.04 ± 0.05,  *z* = - 0.89, *p* > .250 | *M* = -0.02 ± 0.04,  *z* = -0.62, *p* > .250 |
| ‘Social’ conditions learning rate (α_S_) | *M* = - 0.01 ± 0.01,  *z* = - 1.34, *p* = .179 | *M* = - 0.02 ± 0.01,  *z* = - 2.32, *p* = .020 | *M* = - 0.02 ± 0.01,  *z* = -2.66, *p* = .008 |
| Imitation parameter (κ) | *M* = 0.00 ± 0.01,  *z* = 0.02, *p* > .250 | *M* = - 0.00 ± 0.00,  *z* = - 1.08, *p* > .250 | *M* = -0.00 ± 0.00,  *z* = - 0.71, *p* > .250 |
| Social learning parameter (α_O_) | *M* = -0.02 ± 0.01,  *z* = -1.46, *p* = .143 | *M* = - 0.00 ± 0.01,  *z* = -0.41, *p* > .250 | *M* = -0.01 ± 0.01,  *z* = -1.34, *p* = .179 |
