# Supplementary material for: Depressive symptoms are associated with blunted reward learning in social contexts
Source: PLoS Comput Biol. 2019 Jul 29;15(7):e1007224. doi: 10.1371/journal.pcbi.1007224 (PMC6699715; doi:10.1371/journal.pcbi.1007224)
Supplement: S3 Table — (DOCX) [file pcbi.1007224.s003.docx]

**Table S3** – Effect of depression scores on the simulated probabilities of choosing the most rewarded symbol in each sample (mixed linear regression)

|  | Discovery sample | Replication sample | Meta-analysis |
| --- | --- | --- | --- |
| simulated Private condition | *M* = 0.00 ± 0.01,  *t*(46) = 0.43, *p* > .250 | *M* = - 0.01 ± 0.01, *t*(46) = - 1.01, *p* >.250 | *M* = -0.00 ± 0.01,  *z* = -0.29, *p* > .250 |
| Simulated Difference ‘Social Choice’ vs ‘Private’ | *M* = - 0.00 ± 0.02,  *t*(239) = -2.43, *p* = .016 | *M* = - 0.00 ± 0.00,  *t*(239) = -1.28, *p* = .201 | *M* = -0.00 ± 0.00,  *z* = 2.72, *p* = .007 |
| Simulated Difference ‘Social Choice + Outcome’ vs ‘Private’ | *M* = - 0.01 ± 0.01,  *t*(239) = - 1.59, *p* = .113 | *M* = - 0.00 ± 0.00,  *t*(239) = -0.78, *p* > .250 | *M* = - 0.00 ± 0.00,  *z* = -1.74, *p* = .082 |
