# Supplementary material for: Depressive symptoms are associated with blunted reward learning in social contexts
Source: PLoS Comput Biol. 2019 Jul 29;15(7):e1007224. doi: 10.1371/journal.pcbi.1007224 (PMC6699715; doi:10.1371/journal.pcbi.1007224)
Supplement: S4 Table — (DOCX) [file pcbi.1007224.s004.docx]

**Table S4** – Correlation matrix between the model parameters

|  | α_P_ | ß_P_ | α_S_ | ß_S_ | κ | α_O_ |
| --- | --- | --- | --- | --- | --- | --- |
| α_P_ | 1,00 | -0,41*** | 0,22* | -0,10 | -0,02 | 0,17° |
| ß_P_ |  | 1,00 | 0,00 | 0,53*** | -0,05 | -0,23* |
| α_S_ |  |  | 1,00 | -0,19° | -0,16 | 0,18° |
| ß_S_ |  |  |  | 1,00 | 0,06 | -0,37*** |
| κ |  |  |  |  | 1,00 | -0,09 |
| α_O_ |  |  |  |  |  | 1,00 |

Statistical significance: *** p < .001, ** p < .01, * p < .05, ° p < .10
