# Supplementary material for: Depressive symptoms are associated with blunted reward learning in social contexts
Source: PLoS Comput Biol. 2019 Jul 29;15(7):e1007224. doi: 10.1371/journal.pcbi.1007224 (PMC6699715; doi:10.1371/journal.pcbi.1007224)
Supplement: S5 Table — (DOCX) [file pcbi.1007224.s005.docx]

**Table S5** – Meta-analytic model with reversal as cofactor

| **Effect** | **Coefficient** | **SEM** | **z-value** | **P-value** |
| --- | --- | --- | --- | --- |
| Intercept | 0.15 | 0.05 | 5.12 | <.001*** |
| Social-Choice | -0.01 | 0.04 | -1.16 | .872 |
| Social-Choice+Outcome | -0.04 | 0.04 | -1.01 | .315 |
| Demonstrator performance | -0.01 | 0.08 | -0.18 | .860 |
| Trustworthiness | 0.03 | 0.03 | 0.90 | .370 |
| Depressive symptoms | 0.00 | 0.01 | 0.51 | .610 |
| Anxiety symptoms | -0.02 | 0.00 | -0.41 | .686 |
| Reversal | -0.02 | 0.02 | -1.10 | .270 |
| Social-Choice x Demonstrator performance | 0.22 | 0.09 | 2.48 | .013* |
| Social-Choice+Outcome x Demonstrator performance | 0.23 | 0.11 | 2.06 | .040* |
| Social-Choice x Trustworthiness | 0.05 | 0.03 | 1.51 | .131 |
| Social-Choice+Outcome x Trustworthiness | 0.06 | 0.05 | 1.33 | .184 |
| Social-Choice x Depressive symptoms | -0.01 | 0.00 | -2.89 | .004** |
| Social-Choice+Outcome x Depressive symptoms | -0.00 | 0.00 | -0.84 | .396 |
| Social-Choice x Anxiety symptoms | 0.00 | 0.00 | 0.51 | .614 |
| Social-Choice+Outcome x Anxiety symptoms | -0.01 | 0.00 | -0.85 | .395 |
| Social-Choice x Reversal | -0.02 | 0.03 | -0.77 | .441 |
| Social-Choice+Outcome x Reversal | 0.03 | 0.03 | 1.20 | .229 |
