# Supplementary material for: Depressive symptoms are associated with blunted reward learning in social contexts
Source: PLoS Comput Biol. 2019 Jul 29;15(7):e1007224. doi: 10.1371/journal.pcbi.1007224 (PMC6699715; doi:10.1371/journal.pcbi.1007224)
Supplement: S6 Table — (DOCX) [file pcbi.1007224.s006.docx]

**Table S6** – Parameter recovery - Correlation between the recovered parameters and the Depressive symptoms scores for each parameter manipulation

|  | Parameter recovered by fit | | | | | | |
| --- | --- | --- | --- | --- | --- | --- | --- |
| Manipulated parameter |  | α_P_ | ß_P_ | α_S_ | ß_S_ | κ | α_O_ |
|  | α_P_ | **0.6396** | 0.1465 | 0.0212 | 0.0033 | -0.0068 | -0.0068 |
|  | ß_P_ | -0.0282 | **0.7948** | -0.0131 | -0.0152 | -0.0068 | 0.0148 |
|  | α_S_ | 0.0070 | -0.0155 | **0.8448** | 0.0192 | -0.0097 | 0.0139 |
|  | ß_S_ | -0.0065 | 0.0060 | 0.0648 | **0.7351** | 0.0230 | -0.0744 |
|  | κ | -0.0084 | 0.0036 | -0.0174 | 0.0027 | **0.6760** | -0.0078 |
|  | α_O_ | -0.0200 | 0.0085 | 0.0491 | -0.0458 | -0.0075 | **0.7264** |
