# Supplementary figures and images for: Depressive symptoms are associated with blunted reward learning in social contexts
Source: PLoS Comput Biol. 2019 Jul 29;15(7):e1007224. doi: 10.1371/journal.pcbi.1007224 (PMC6699715; doi:10.1371/journal.pcbi.1007224)

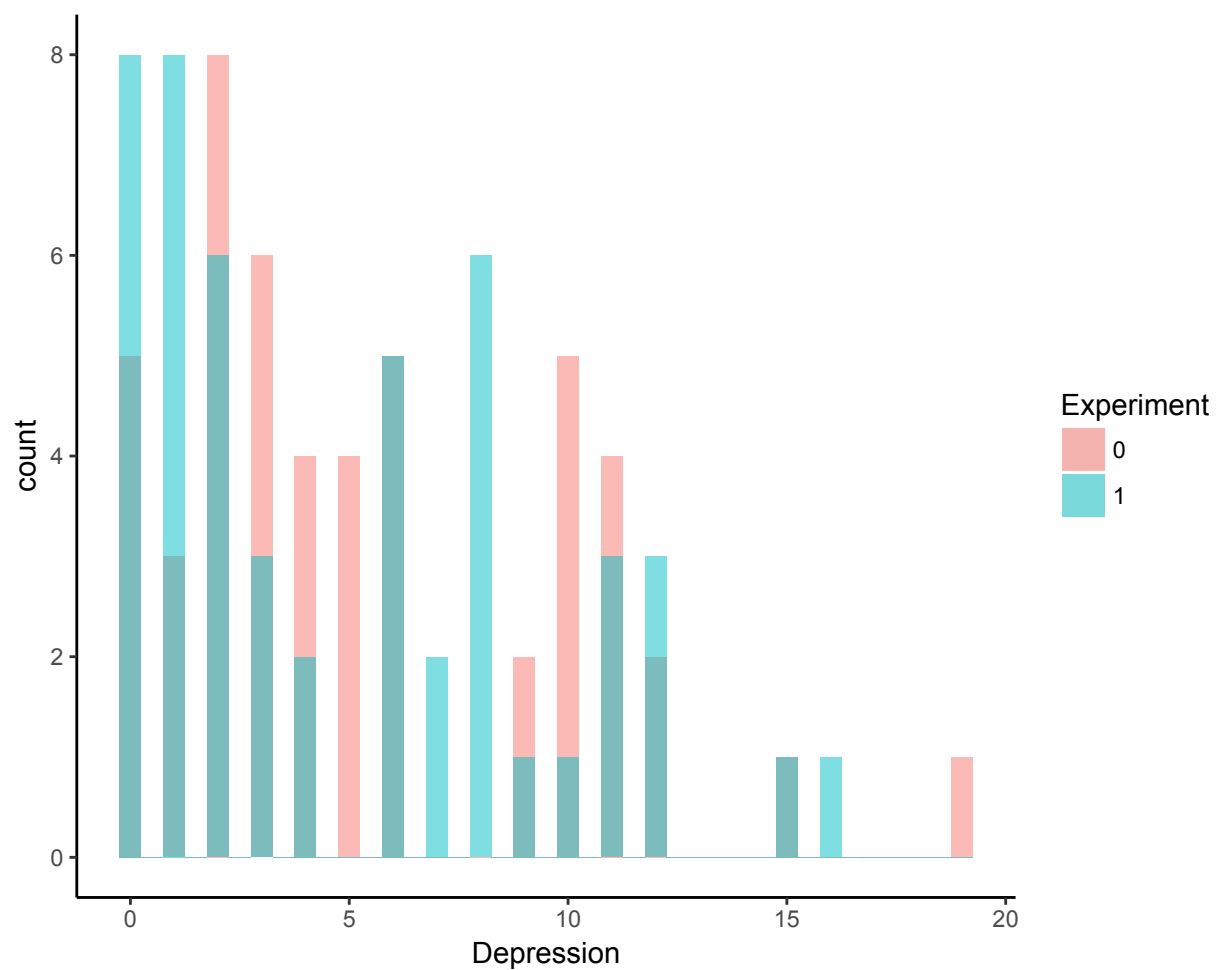

Supplement: S1 Fig — (PDF) [file pcbi.1007224.s007.pdf]

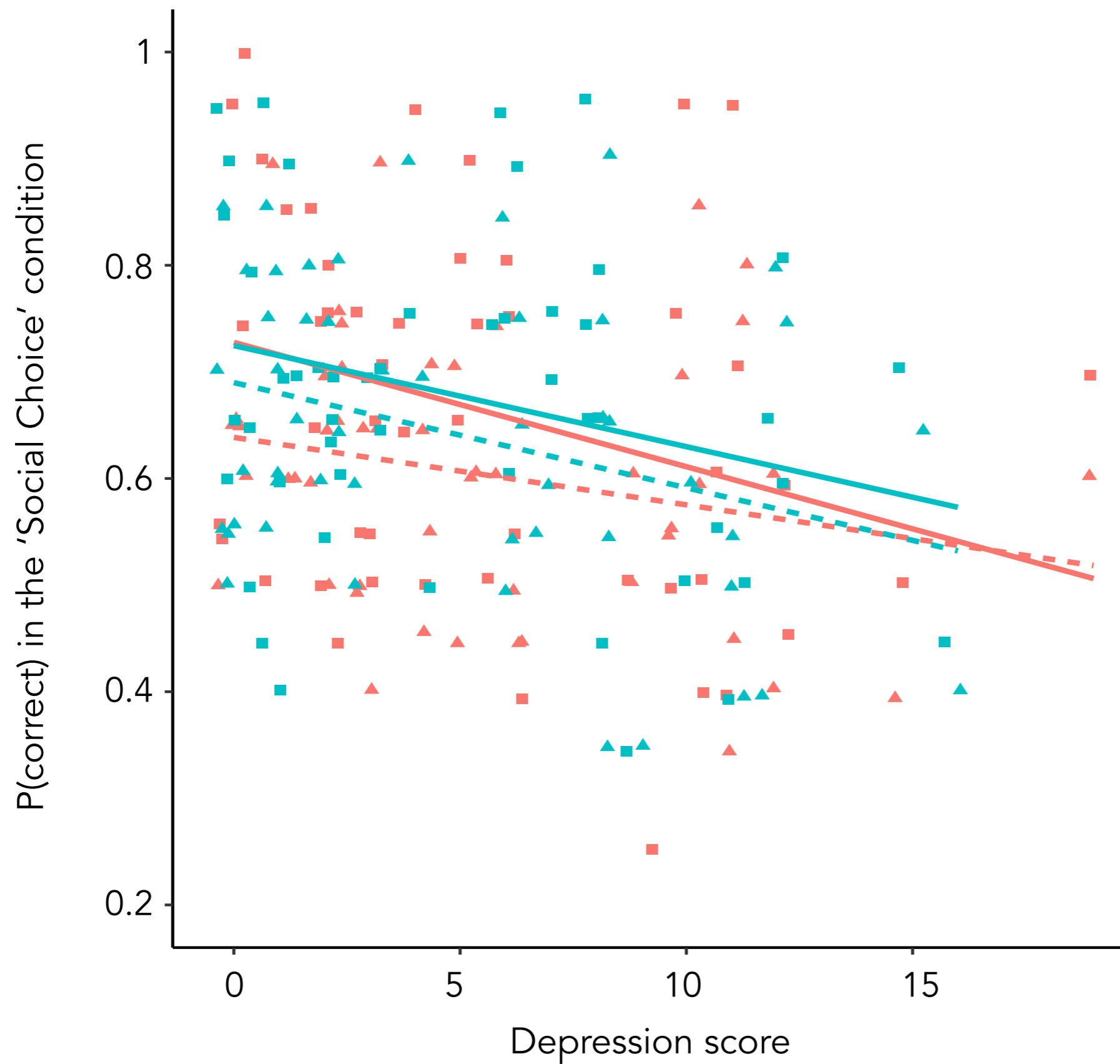

● Discovery sample    ● Replication sample    — Stable blocks    - - - Reversal blocks

Supplement: S2 Fig — (PDF) [file pcbi.1007224.s008.pdf]

**'Private'**

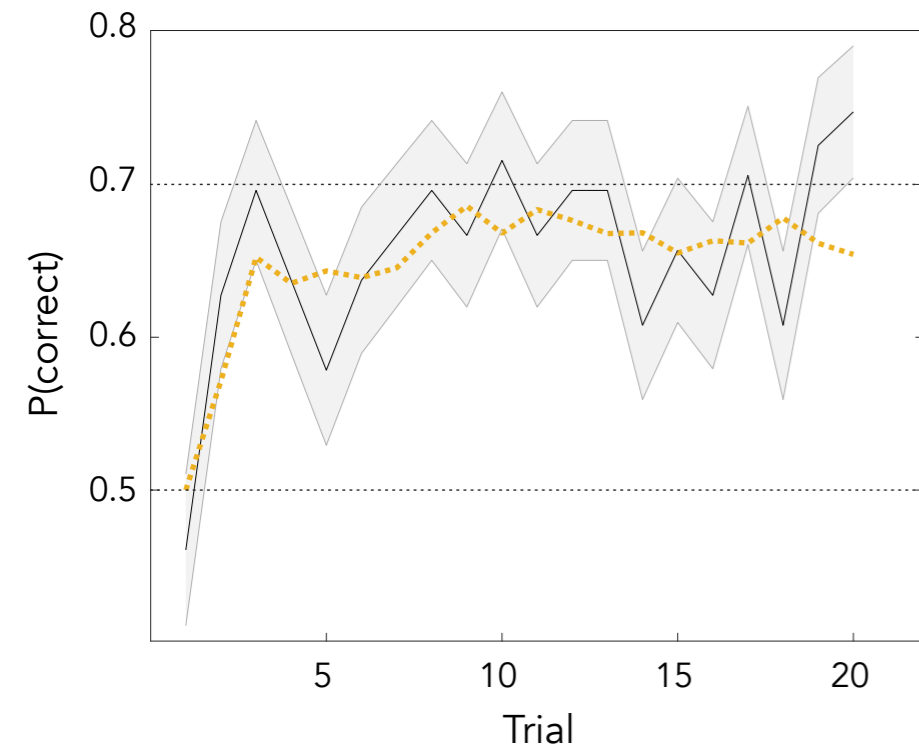

**'Social Choice'**

Stable blocks

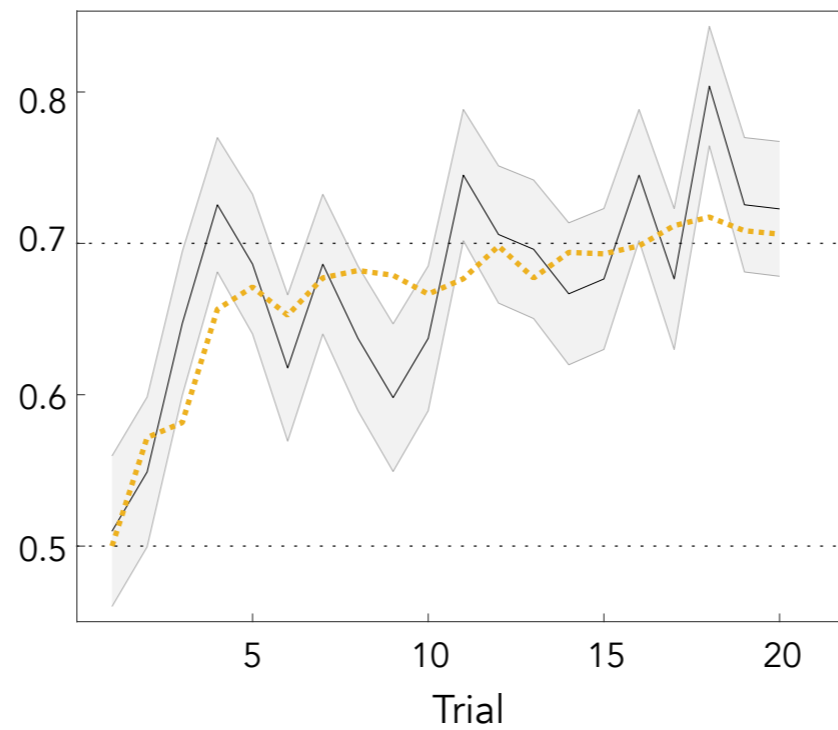

**'Social Choice+Outcome'**

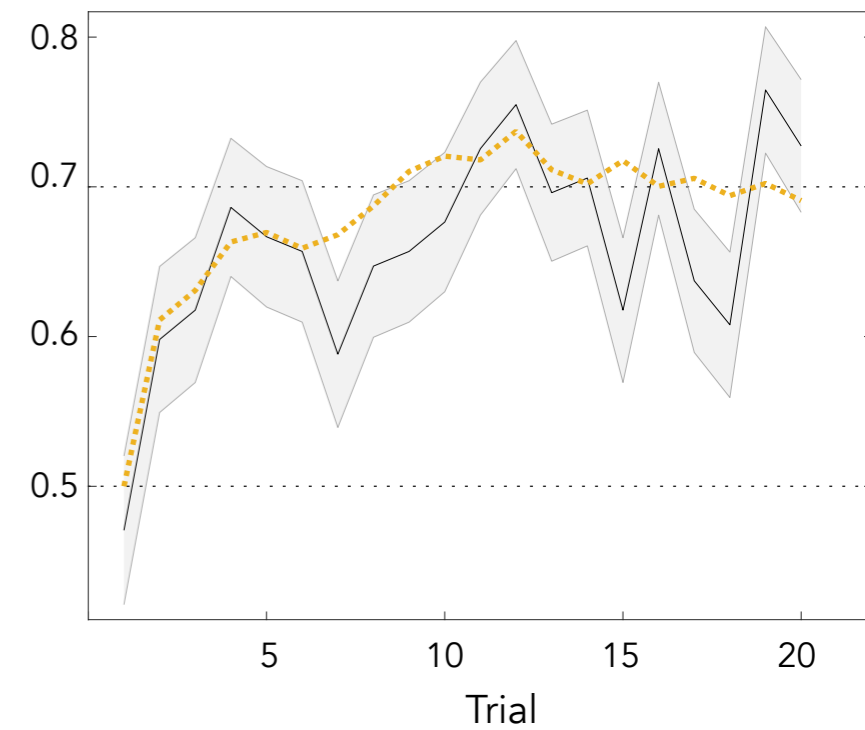

Reversal blocks

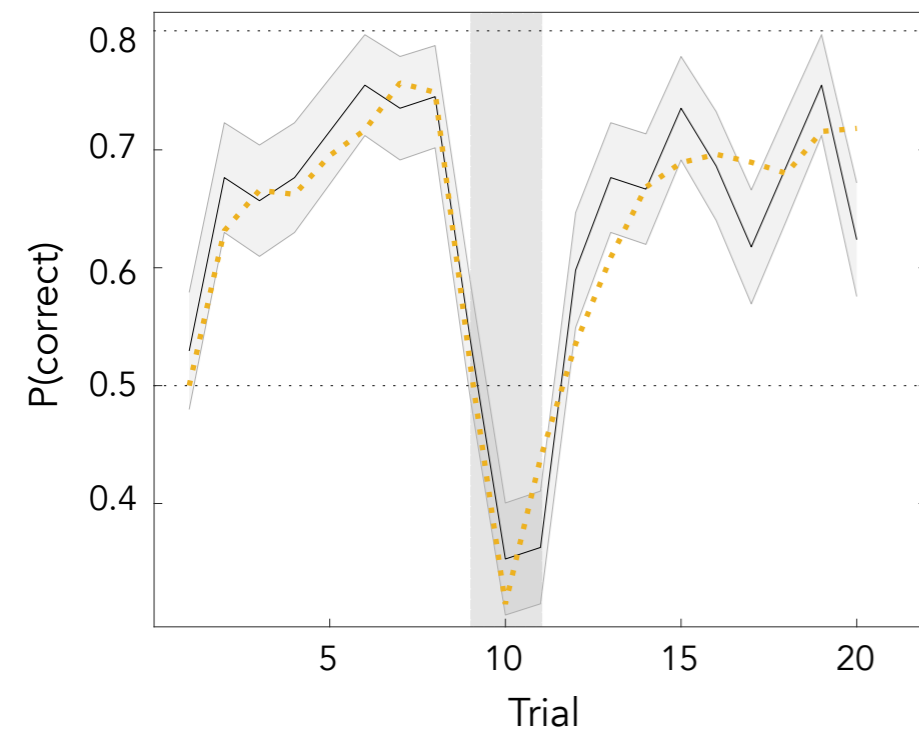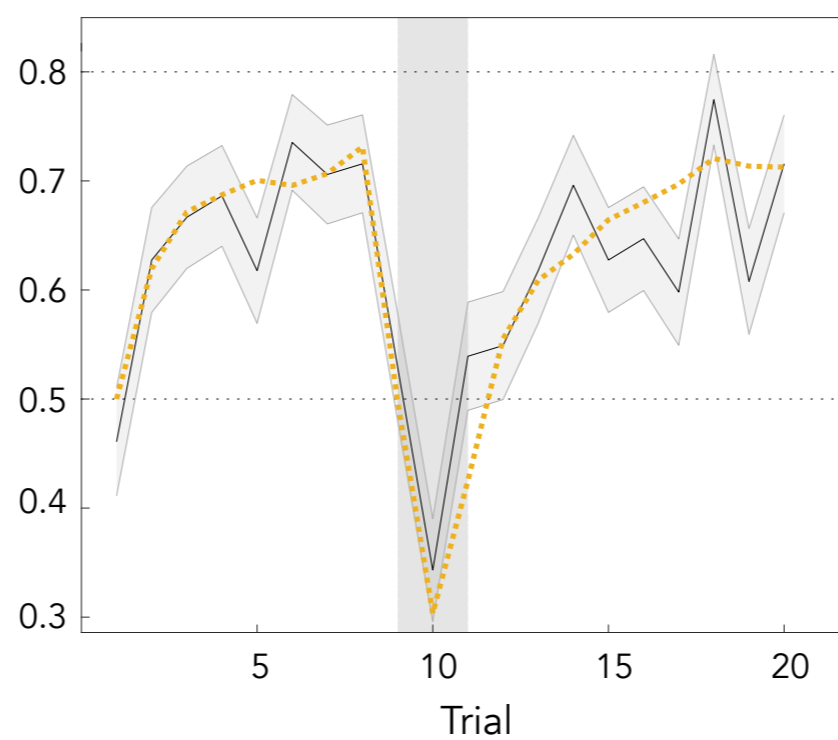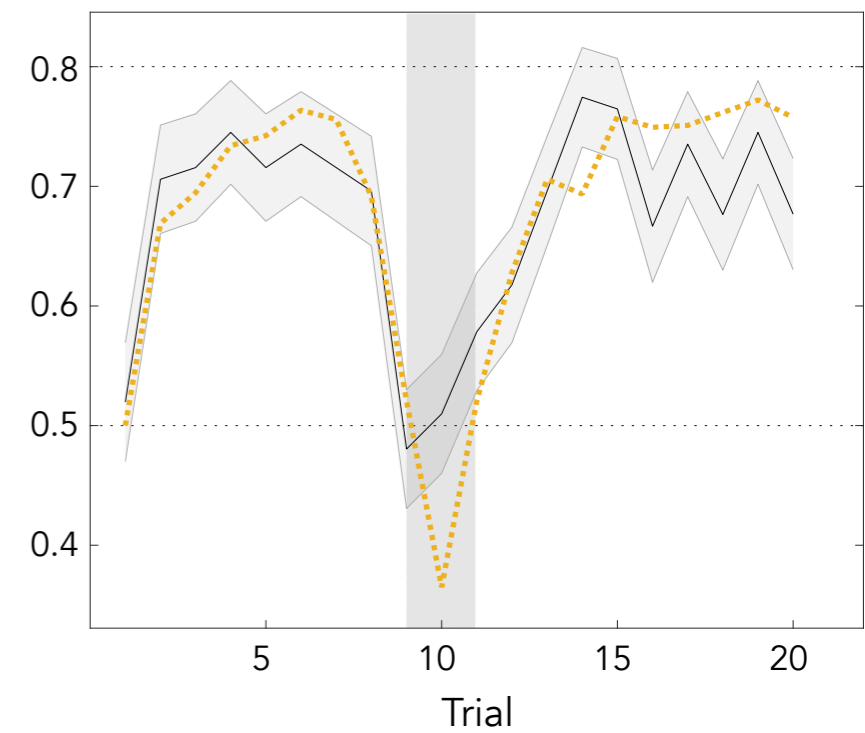

Supplement: S3 Fig — Mean learning curves (in black) and their standard errors (shaded light grey area) are represented for each condition and each reward contingency. The dotted line in orange represents the model prediction for each condition and each reward contingency. The grey area for the reversal blocks indicates the trials in each the reversal of reward contingencies can occur. For each plot, the top dotted line indicates the matching law and the bottom dotted line indicates chance level. (PDF) [file pcbi.1007224.s009.pdf]

● Discovery sample

● Replication sample

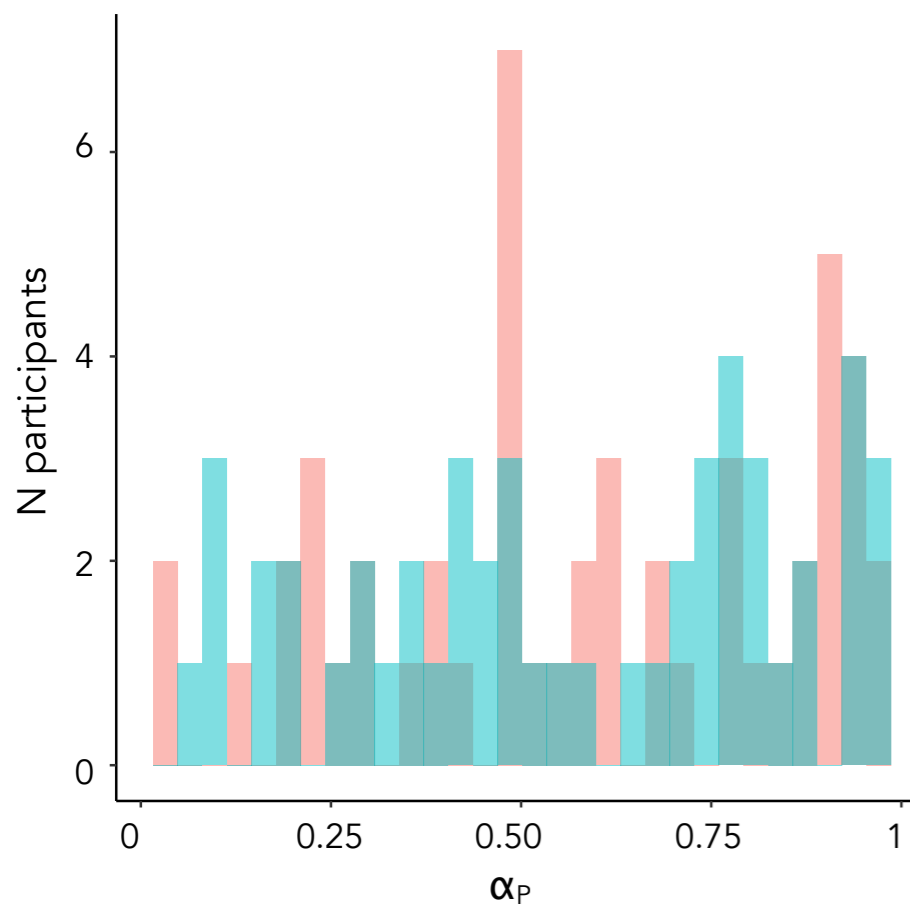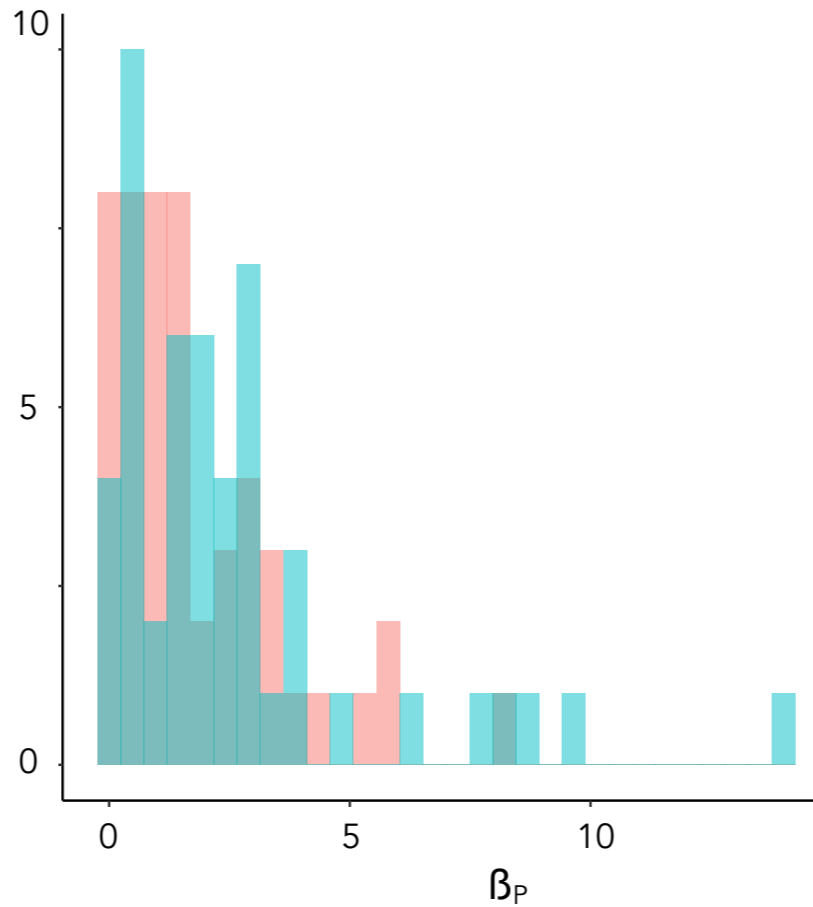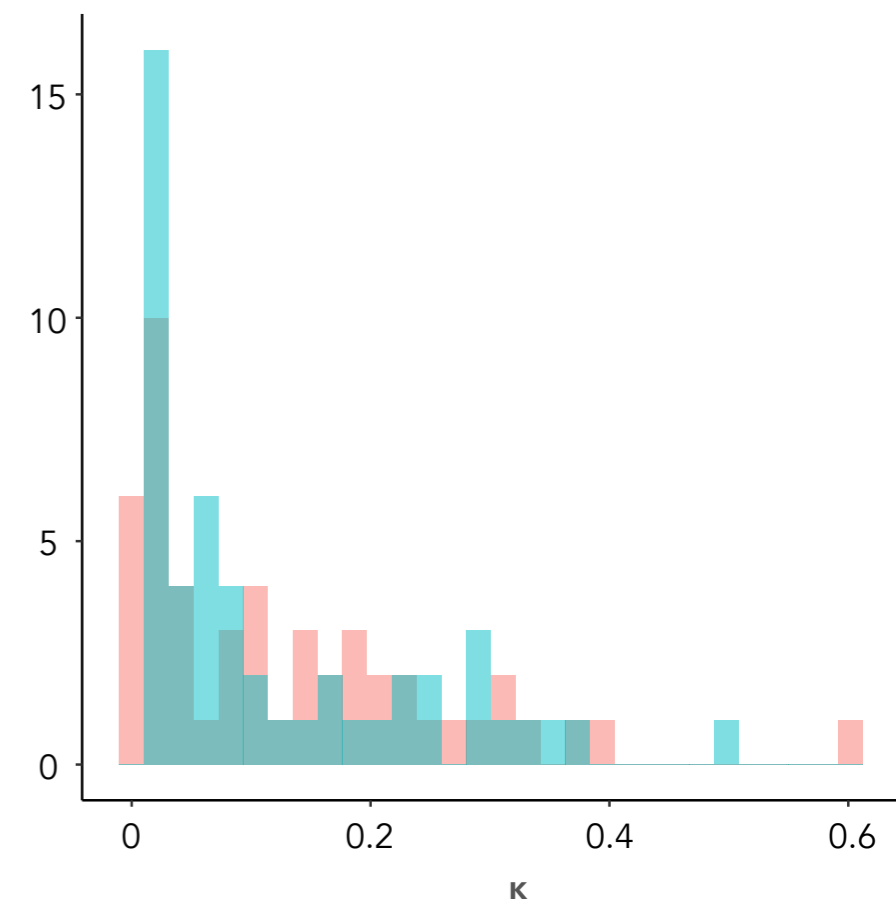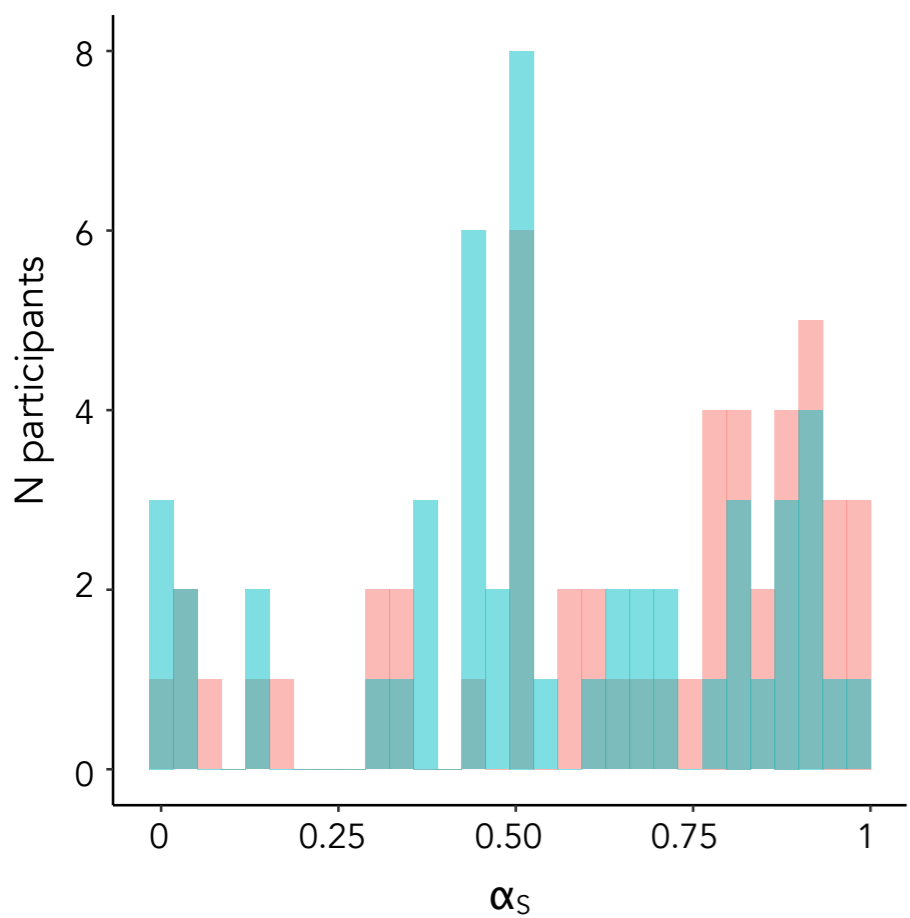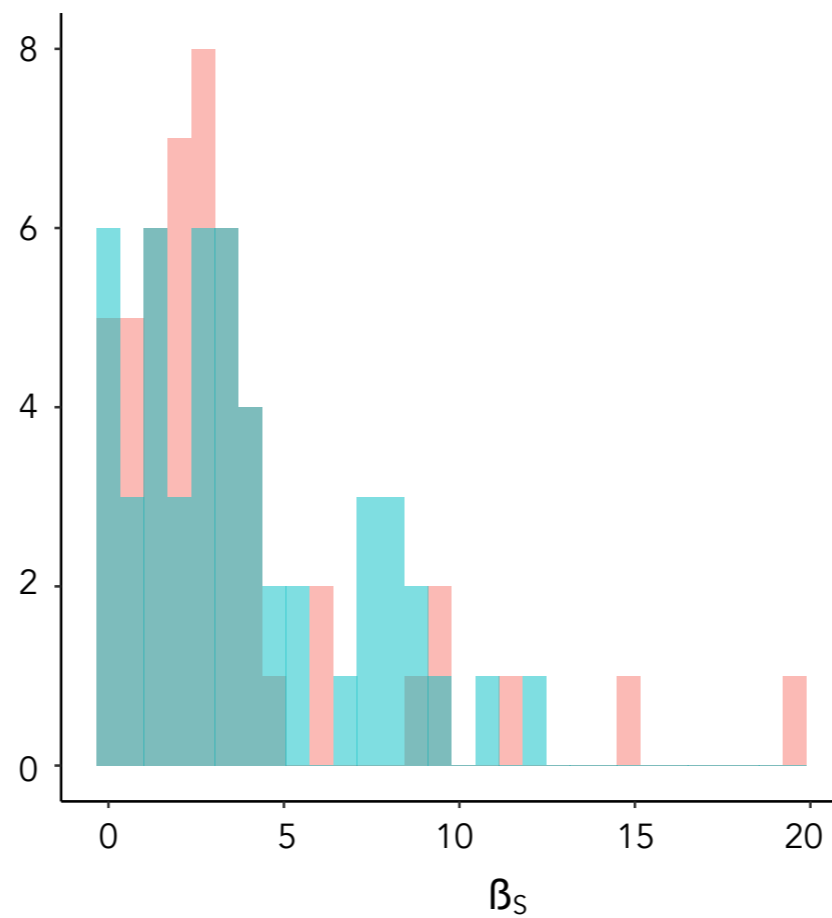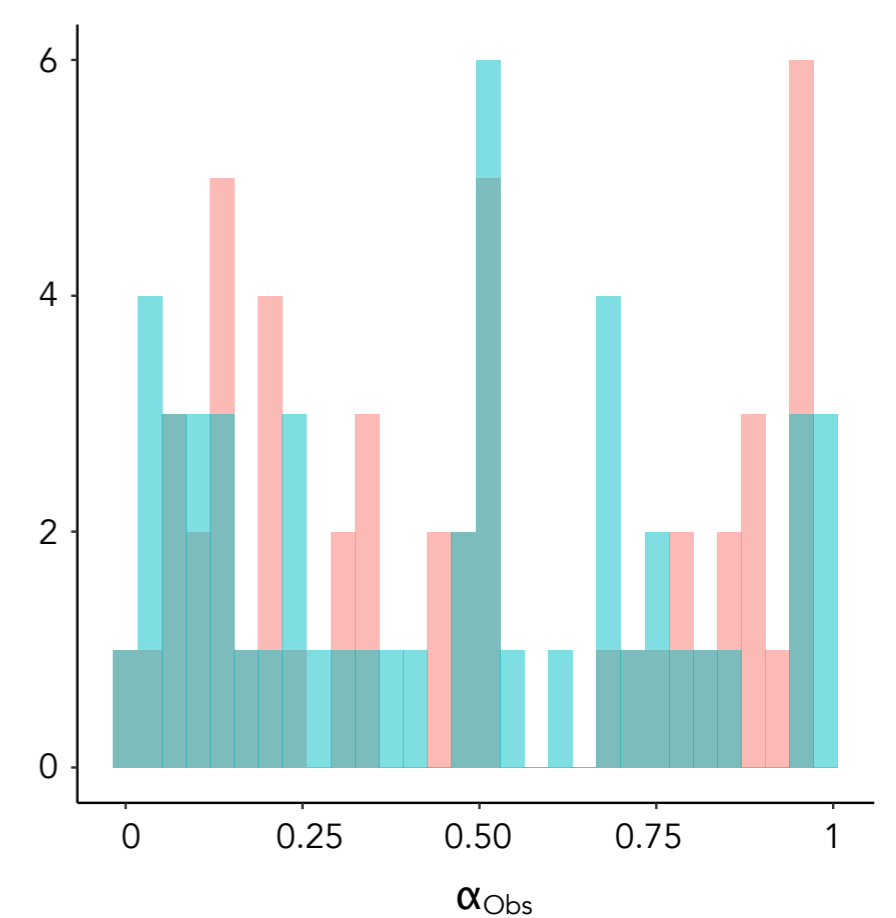

Supplement: S5 Fig — (PDF) [file pcbi.1007224.s011.pdf]

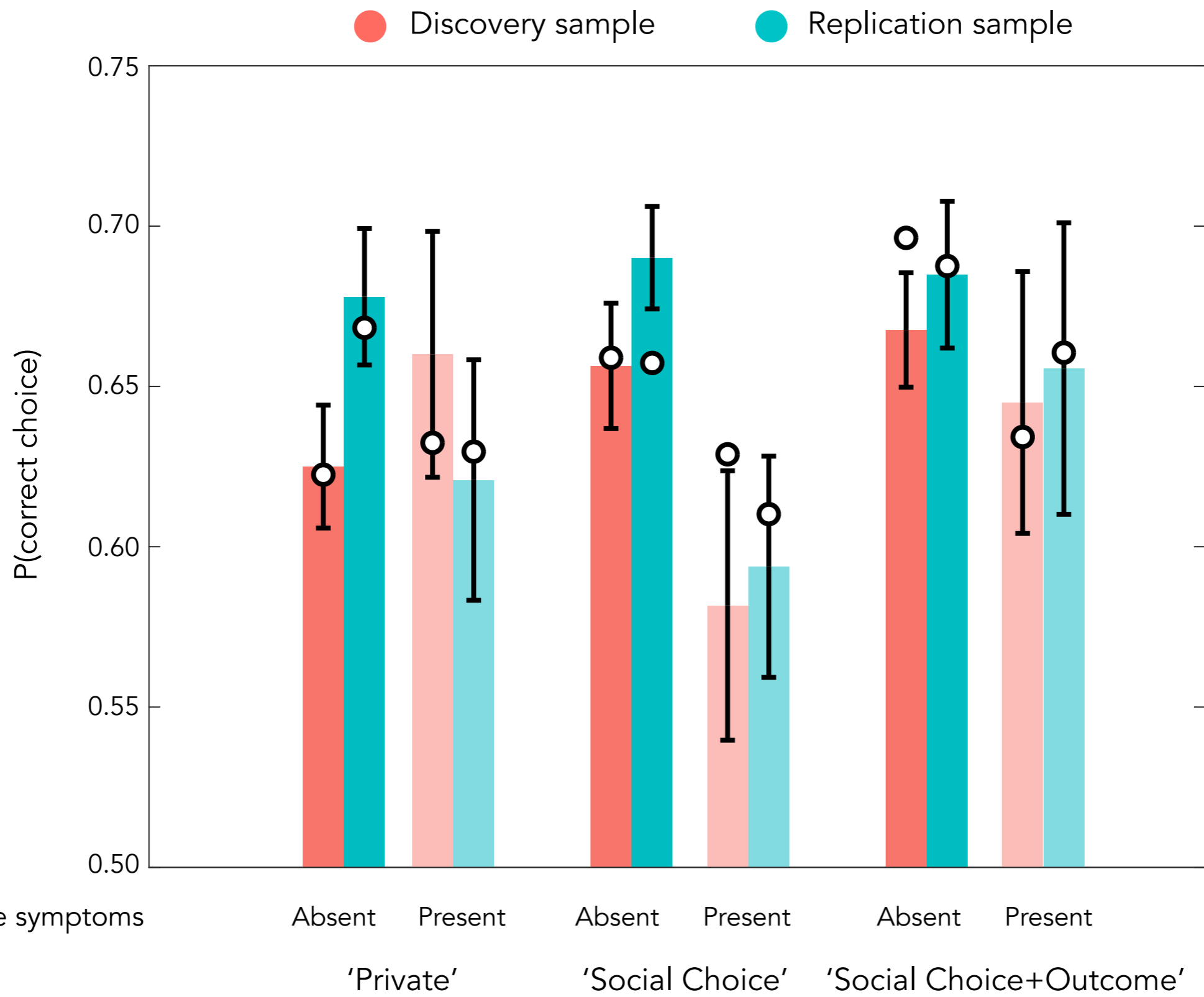

Supplement: S6 Fig — Depressive symptoms (HAD Depression subscale score ≥ 8) were associated with decreased correct response rate only in the ‘Social Choice’ condition. This effect was accurately recovered by simulations of our model (white dots). Error bars represent standard errors. (PDF) [file pcbi.1007224.s012.pdf]

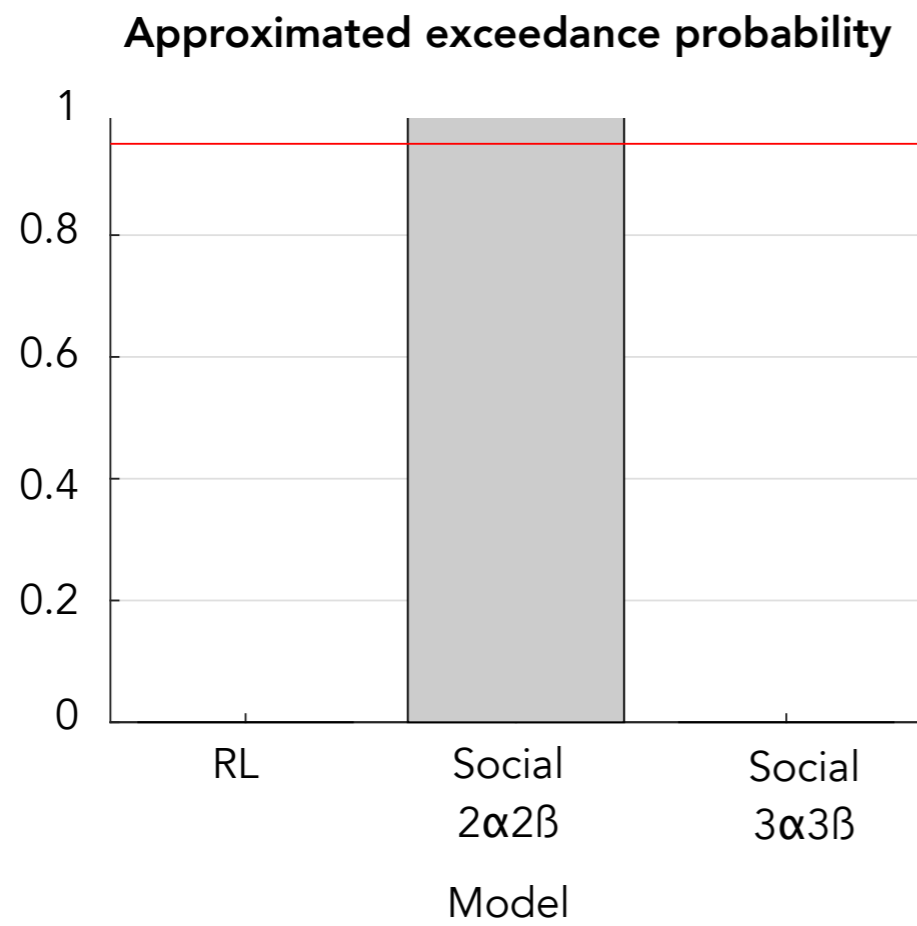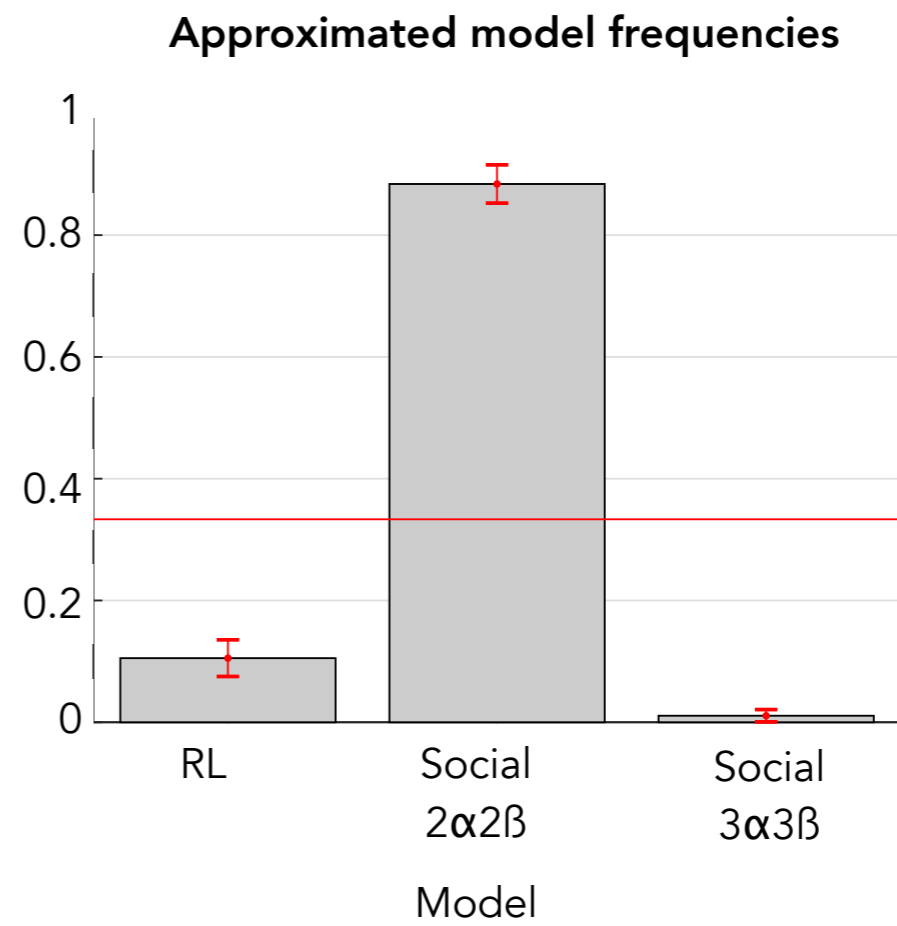

Supplement: S8 Fig — In order to further test the robustness of our results we first compared our model with a more complex model including different learning rates and temperature parameters for each condition. This parsimony-driven model comparison including this model confirmed that the one we used in our analyses better accounted our data. We then compared our model with all the models of the possible models containing one to three learning rates and one to three temperature parameters or two temperature parameters in addition to the imitation (κ) and the observation learning rate parameter (αO) and a simple reinforcement learning model. In line with our results, the model with two learning parameters and one temperature parameter was the most probable for our data (S9 Fig). In addition, we recovered the specific association between higher depression scores and lower learning rates in the social conditions with the learning parameters estimated in this model (b = -0.2 ± 0.01, z = -2.55, p = .011, all other |z| < 1.48, all p-s > .137; S10 Fig). (PDF) [file pcbi.1007224.s014.pdf]

**Approximated exceedance probability**

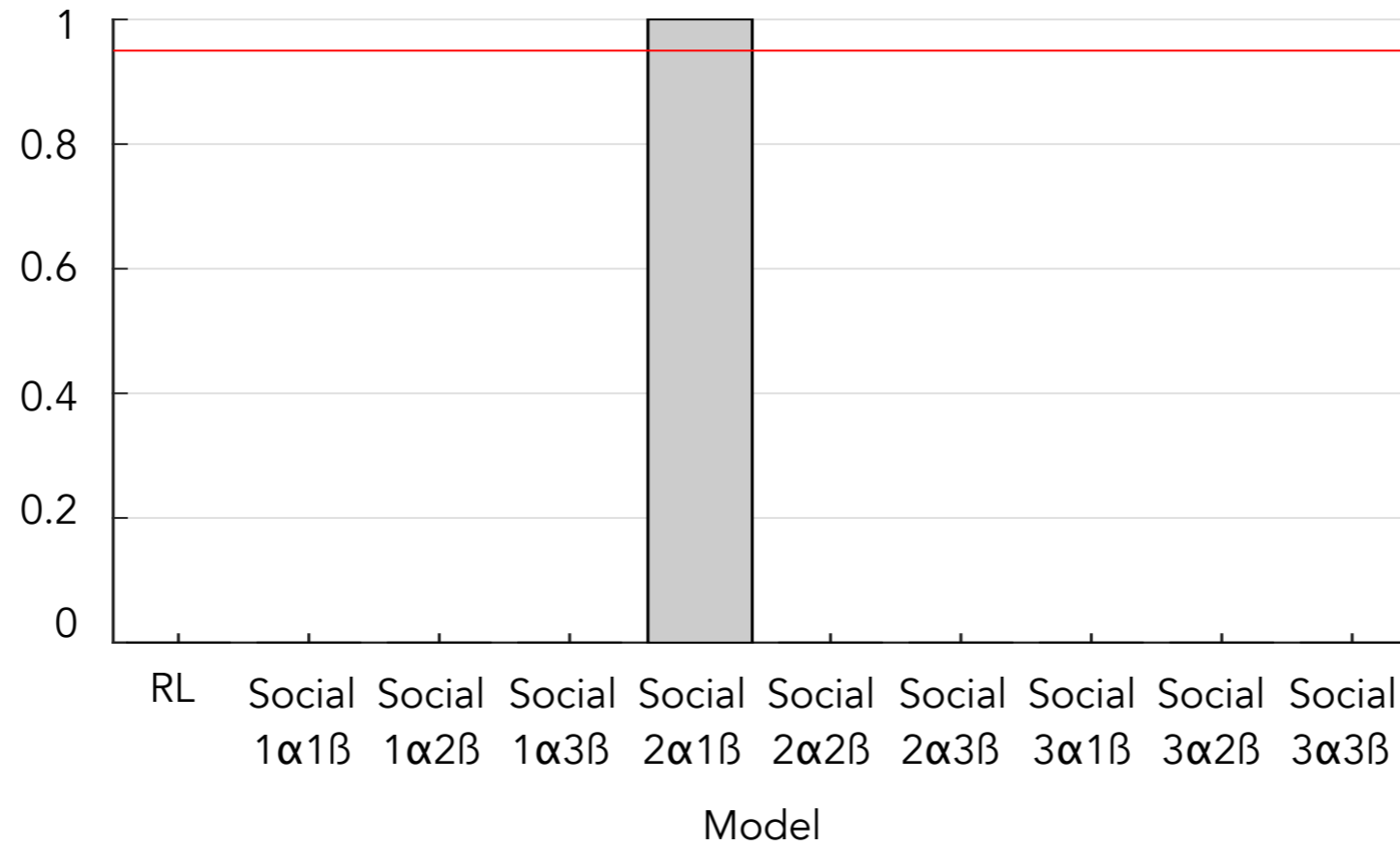

**Approximated model frequencies**

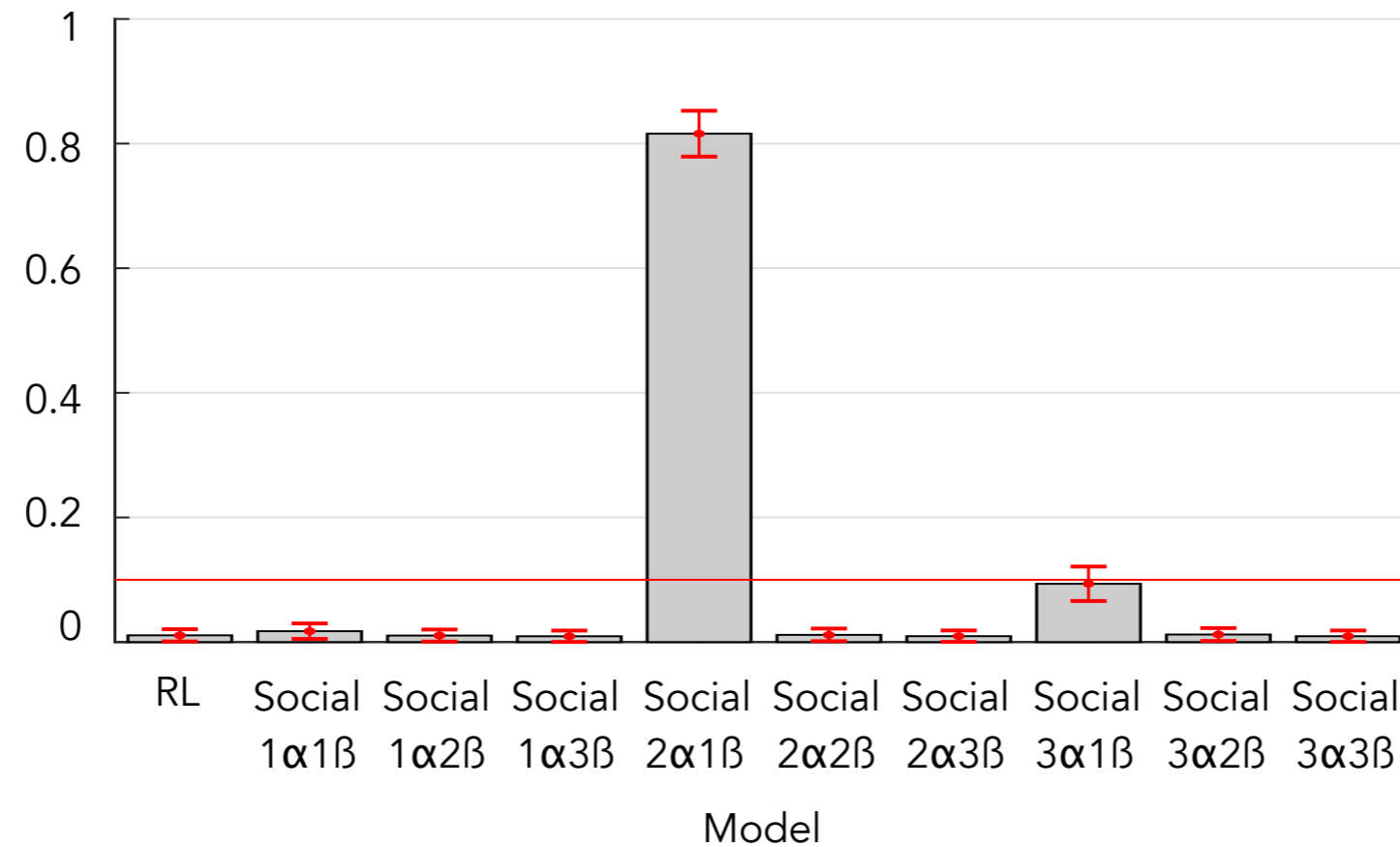

Supplement: S9 Fig — (PDF) [file pcbi.1007224.s015.pdf]
