# Supplementary material for: Depressive symptoms are associated with blunted reward learning in social contexts
Source: PLoS Comput Biol. 2019 Jul 29;15(7):e1007224. doi: 10.1371/journal.pcbi.1007224 (PMC6699715; doi:10.1371/journal.pcbi.1007224)
Supplement: S4 Fig — For each of the condition, the performance predicted by the computational model highly correlated with the participants’ actual performances in both the discovery and the replication samples (Meta-analytic correlations: all r-s > .72, all z-s > 10.21, all p-s < .001). (PDF) [file pcbi.1007224.s010.pdf]

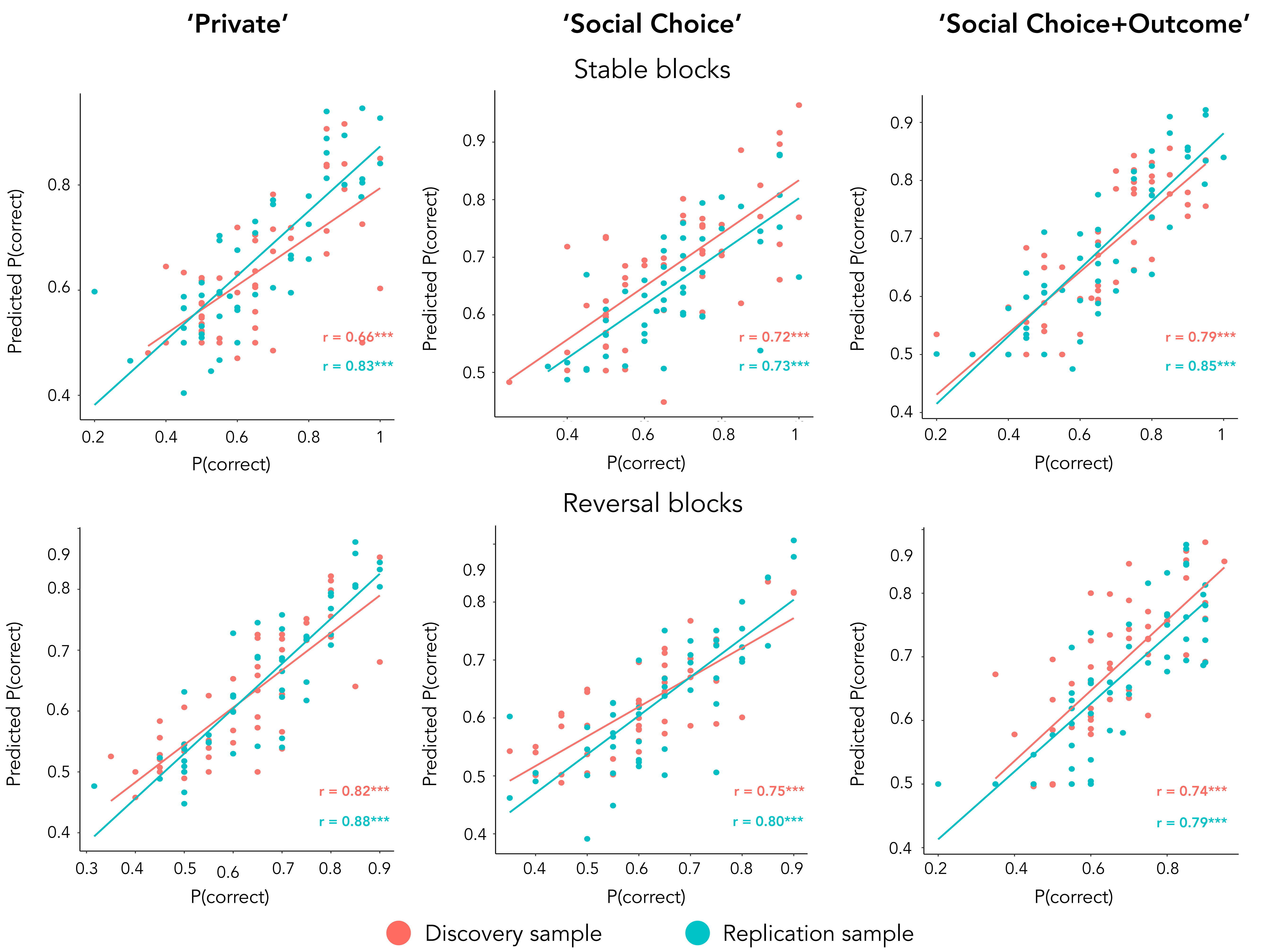

'Private'
'Social Choice'
'Social Choice+Outcome'
Stable blocks
Reversal blocks
Predicted P(correct)
P(correct)
r = 0.66***
r = 0.83***
r = 0.72***
r = 0.73***
r = 0.79***
r = 0.85***
r = 0.82***
r = 0.88***
r = 0.75***
r = 0.80***
r = 0.74***
r = 0.79***
Discovery sample
Replication sample
