# Supplementary material for: Depressive symptoms are associated with blunted reward learning in social contexts
Source: PLoS Comput Biol. 2019 Jul 29;15(7):e1007224. doi: 10.1371/journal.pcbi.1007224 (PMC6699715; doi:10.1371/journal.pcbi.1007224)
Supplement: S7 Fig — The correlation coefficients of the two samples were highly correlated, indicating the replication of the results in the two samples. The dotted line corresponds to the perfect replication. (PDF) [file pcbi.1007224.s013.pdf]

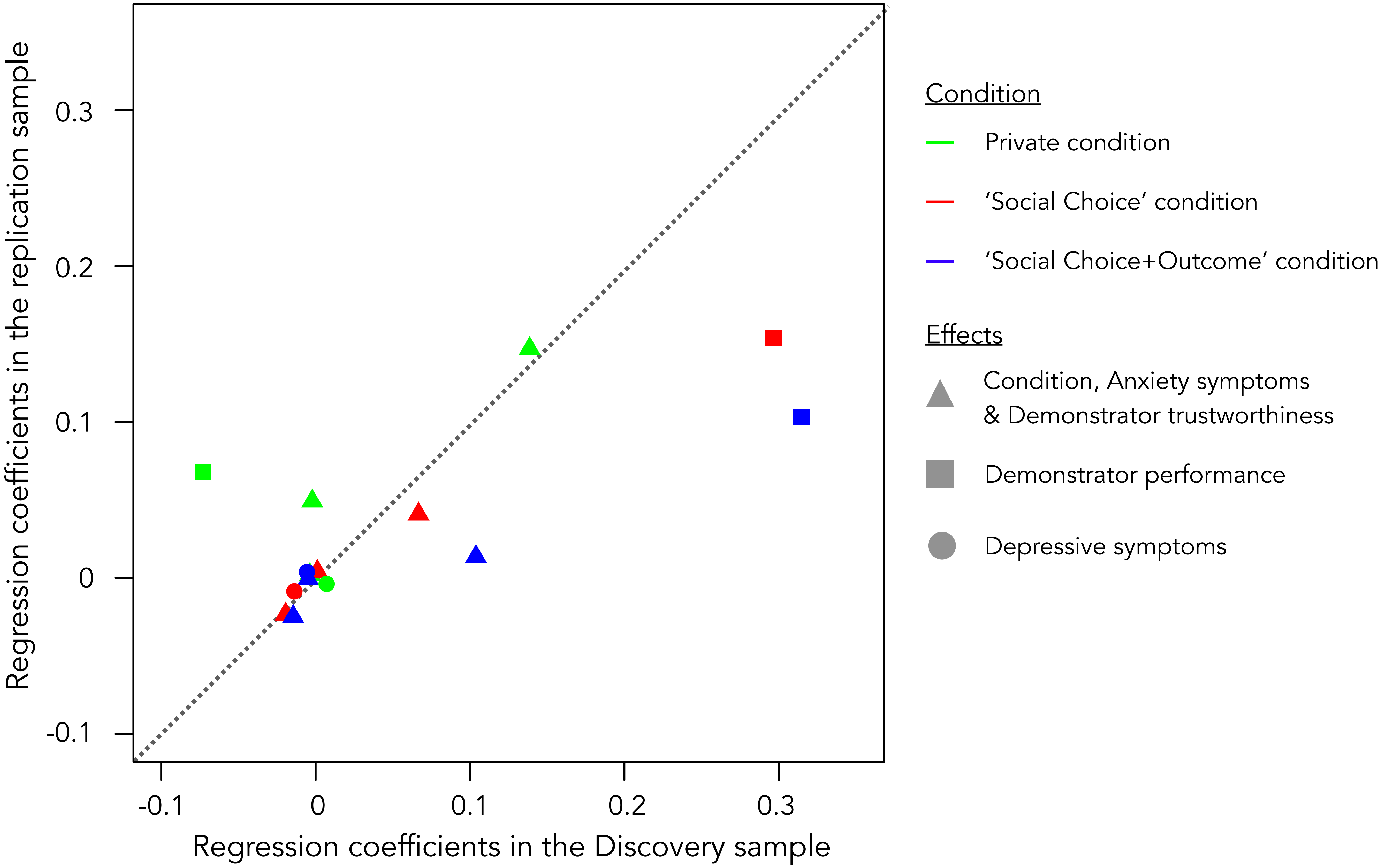
Regression coefficients in the replication sample
0.3
0.2
0.1
0
-0.1
-0.1
0
0.1
0.2
0.3
Regression coefficients in the Discovery sample
Condition
Private condition
'Social Choice' condition
'Social Choice+Outcome' condition
Effects
Condition, Anxiety symptoms & Demonstrator trustworthiness
Demonstrator performance
Depressive symptoms
