# Supplementary material for: Depressive symptoms are associated with blunted reward learning in social contexts
Source: PLoS Comput Biol. 2019 Jul 29;15(7):e1007224. doi: 10.1371/journal.pcbi.1007224 (PMC6699715; doi:10.1371/journal.pcbi.1007224)
Supplement: S10 Fig — The correlation is also significant (b = -0.2 ± 0.01, z = -2.55, p = .011). (PDF) [file pcbi.1007224.s016.pdf]

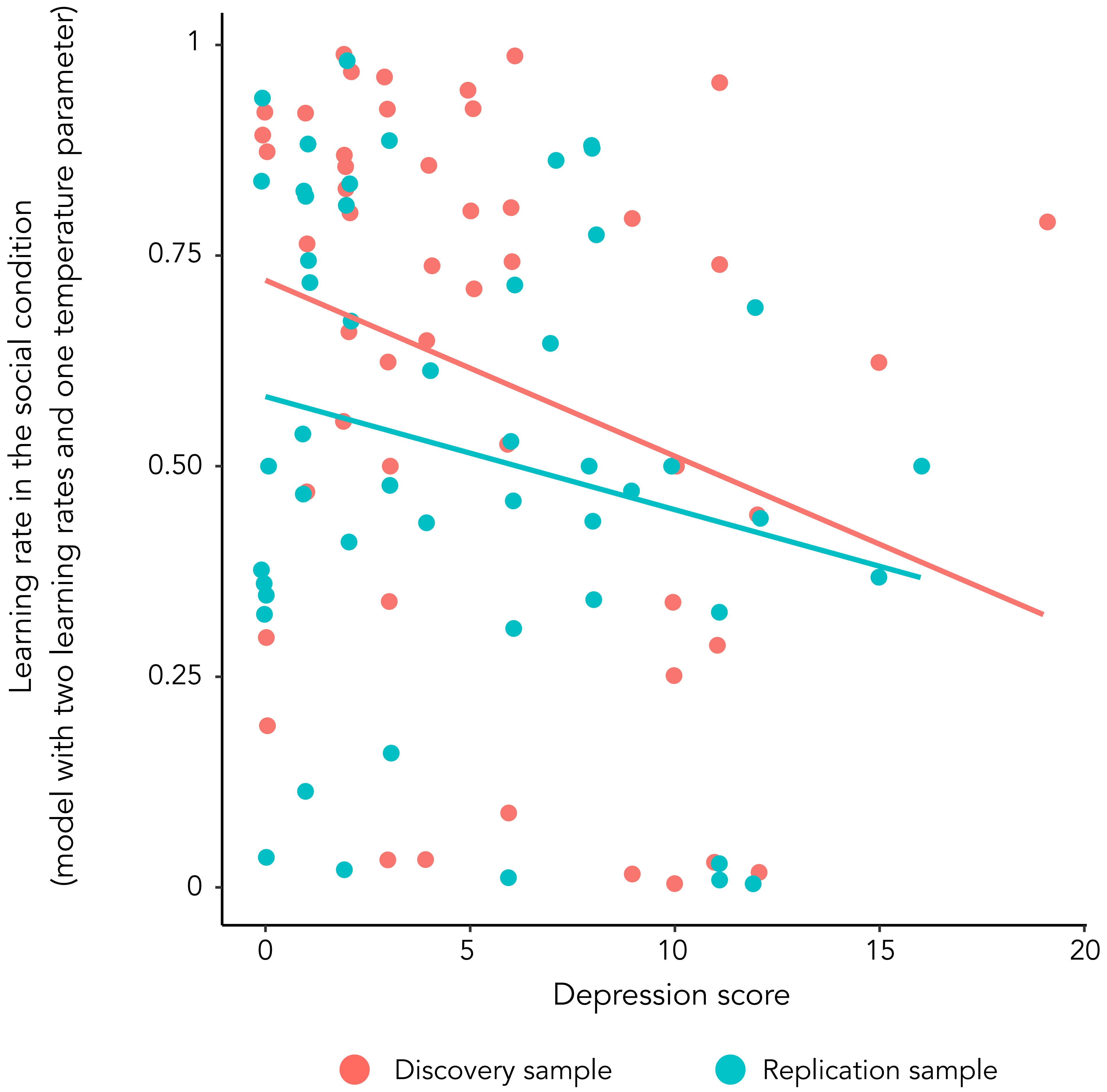

Learning rate in the social condition
(model with two learning rates and one temperature parameter)
1
0.75
0.50
0.25
0
0
5
10
15
20
Depression score
Discovery sample
Replication sample
